# High-dose rifampin improves bactericidal activity without increased intracerebral inflammation in animal models of tuberculous meningitis

**DOI:** 10.1172/JCI155851

**Published:** 2022-03-15

**Authors:** Camilo A. Ruiz-Bedoya, Filipa Mota, Elizabeth W. Tucker, Farina J. Mahmud, Maria I. Reyes-Mantilla, Clara Erice, Melissa Bahr, Kelly Flavahan, Patricia de Jesus, John Kim, Catherine A. Foss, Charles A. Peloquin, Dima A. Hammoud, Alvaro A. Ordonez, Carlos A. Pardo, Sanjay K. Jain

**Affiliations:** 1 Center for Infection and Inflammation Imaging Research,; 2 Center for Tuberculosis Research,; 3 Department of Pediatrics,; 4 Department of Anesthesiology and Critical Care Medicine,; 5 Department of Neurology, and; 6 Russell H. Morgan Department of Radiology and Radiological Sciences, Johns Hopkins University School of Medicine, Baltimore, Maryland, USA.; 7 Infectious Disease Pharmacokinetics Laboratory, Pharmacotherapy and Translational Research, University of Florida College of Pharmacy, Gainesville, Florida, USA.; 8 Center for Infectious Disease Imaging, Radiology and Imaging Sciences, NIH, Bethesda, Maryland, USA.; 9 Department of Pathology, Johns Hopkins University School of Medicine, Baltimore, Maryland, USA.

**Keywords:** Infectious disease, Microbiology, Neuroimaging, Neurological disorders, Pharmacology

## Abstract

Tuberculous meningitis (TB meningitis) is the most severe form of tuberculosis (TB), requiring 12 months of multidrug treatment for cure, and is associated with high morbidity and mortality. High-dose rifampin (35 mg/kg/d) is safe and improves the bactericidal activity of the standard-dose (10 mg/kg/d) rifampin-containing TB regimen in pulmonary TB. However, there are conflicting clinical data regarding its benefit for TB meningitis, where outcomes may also be associated with intracerebral inflammation. We conducted cross-species studies in mice and rabbits, demonstrating that an intensified high-dose rifampin-containing regimen has significantly improved bactericidal activity for TB meningitis over the first-line, standard-dose rifampin regimen, without an increase in intracerebral inflammation. Positron emission tomography in live animals demonstrated spatially compartmentalized, lesion-specific pathology, with postmortem analyses showing discordant brain tissue and cerebrospinal fluid rifampin levels and inflammatory markers. Longitudinal multimodal imaging in the same cohort of animals during TB treatment as well as imaging studies in two cohorts of TB patients demonstrated that spatiotemporal changes in localized blood-brain barrier disruption in TB meningitis are an important driver of rifampin brain exposure. These data provide unique insights into the mechanisms underlying high-dose rifampin in TB meningitis with important implications for developing new antibiotic treatments for infections.

## Graphical abstract

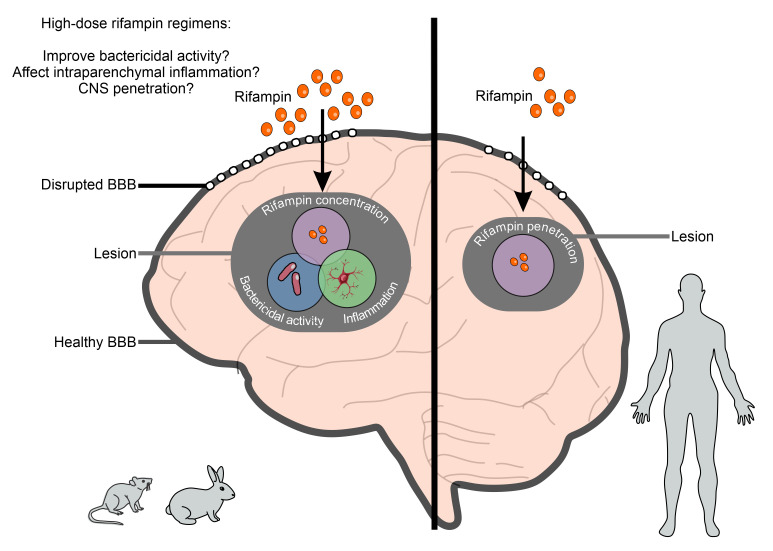

## Introduction

Tuberculous meningitis (TB meningitis) is the most devastating form of tuberculosis (TB), especially among HIV-infected individuals and young children (1–3). Despite the knowledge that key antimicrobials do not penetrate into the brain adequately and that immunopathology is the critical pathologic process, current treatments for TB meningitis are not optimized and continue to be based on those used for pulmonary TB (2).

Rifampin, an inhibitor of DNA-dependent RNA polymerase, has potent, dose-dependent activity against *Mycobacterium tuberculosis*, with the area under the time-concentration curve (AUC) being the most predictive of bactericidal activity (4–7). Higher rifampin doses could be a promising approach to shortening TB treatment (8–11), with doses up to 35 mg/kg/d demonstrated to be safe in adults with pulmonary TB (8, 12) and children with many forms of TB (13). Unfortunately, rifampin has limited penetration into the CNS with current regimens and decreases rapidly after treatment initiation (12, 14), leaving room for advances in dose optimization for improved delivery. Moreover, clinical trials in patients with TB meningitis have demonstrated conflicting results, with intravenous administration of high-dose rifampin (13 mg/kg/d) during the first 2 weeks substantially lowering mortality (35% versus 65% in controls) in one study, though a subsequent larger randomized trial utilizing oral rifampin (15 mg/kg/d) did not support this benefit (13, 15). While differences in rifampin exposures in the two trials could explain these differences, it has also been hypothesized that outcomes in TB meningitis may be more strongly associated with changes in intracerebral inflammation rather than bacterial killing. This is relevant, as intensified TB regimens with enhanced bacterial killing could worsen intracerebral inflammation due to the release of proinflammatory components during bacterial lysis. Therefore, the role of high-dose rifampin in improving treatments for TB meningitis remains unknown.

**Figure 1:**
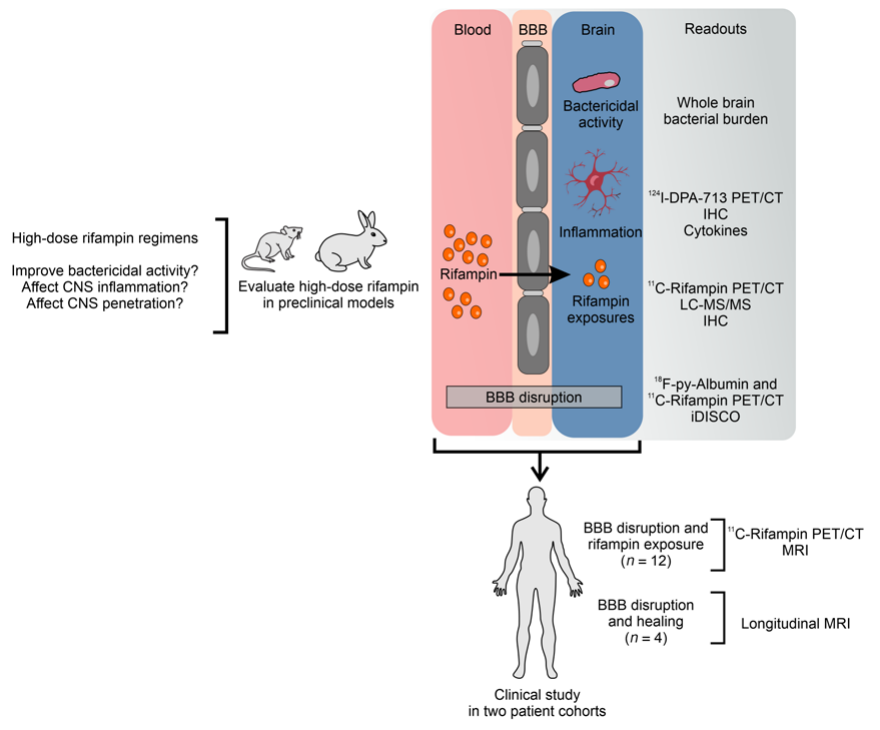
Study design. Cross-species studies were performed in mice, rabbits, and humans to address key questions regarding the use of high-dose rifampin for TB meningitis. Longitudinal, multimodal imaging studies in live animals, supported by postmortem assays, were designed to evaluate the bactericidal activity of high- versus standard-dose rifampin regimens as well as lesion-specific, intracerebral inflammation. Studies to assess intracerebral, lesion-specific rifampin exposures and expression of efflux pumps (e.g., MDR-1) were performed with the goal of identifying pathways that could be modulated to optimize rifampin delivery to the CNS. We also studied the effect of dexamethasone on rifampin levels, intracerebral inflammation, and the efficacy of rifampin-containing regimens. Multimodal imaging in the same cohort of animals was utilized to explore the mechanisms underlying the spatiotemporal changes in lesion-specific, rifampin brain exposures, and localized blood-brain barrier disruptions (^18^F-py-albumin PET/CT). Postmortem analyses to assess vessel pathology at high resolution were also performed (clarified whole mouse brains — iDISCO protocol). Finally, longitudinal imaging studies in 2 cohorts of patients were analyzed to understand the spatiotemporal changes in rifampin exposures and localized blood-brain barrier disruptions during TB treatment and correlated with the findings from the animal studies. LC–MS/MS, liquid chromatography and tandem mass spectrometry; IHC, immunohistochemistry; BBB, blood-brain barrier.

Unlike in pulmonary TB, animal models have not been widely utilized to evaluate new TB drugs and regimens for TB meningitis prior to testing in clinical trials (2), which are not only expensive, but can take several years. Additionally, due to the difficulties and risks of sampling brain tissues, we currently have limited information regarding antimicrobial drug concentrations and intracerebral inflammation at infection sites (where the pathogen resides). Here, we performed cross-species studies to answer key questions regarding the use of high-dose rifampin for TB meningitis (Figure 1). We performed cross-species studies to evaluate a high-dose (35 mg/kg/d) rifampin-containing regimen in what we believe to be a newly developed mouse model and an established rabbit model (16) of TB meningitis. Advanced PET/CT imaging was utilized for noninvasive, longitudinal monitoring of lesion-specific, intracerebral inflammation during TB treatment. ^124^I-DPA-713, a ligand for the translocator protein, was utilized as an imaging biomarker of activated microglia and macrophages in mice, providing a noninvasive readout of neuroinflammation (17–19). Multimodal imaging in the same cohort of animals was utilized to explore the mechanisms underlying the spatiotemporal changes in lesion-specific, rifampin brain exposures (^11^C-rifampin PET/CT) (10, 12) and localized blood-brain barrier disruptions (^18^F-py-albumin PET/CT). Postmortem analyses to assess detailed vascular pathology (in whole clarified brains), tissue and cerebrospinal fluid (CSF) cytokine levels, immunohistochemistry (inflammation, multidrug resistance protein 1 [MDR-1] efflux pump), and drug levels were performed. Finally, longitudinal imaging studies (MRI, ^18^F-fluorodeoxyglucose [FDG] PET/CT, and ^11^C-rifampin PET/CT) and brain biopsies from patients with TB meningitis were analyzed to help us understand the spatiotemporal changes during TB treatments and correlated with the findings from the animal studies.

## Results

### Animal models of TB meningitis recapitulate human disease

**Figure 2:**
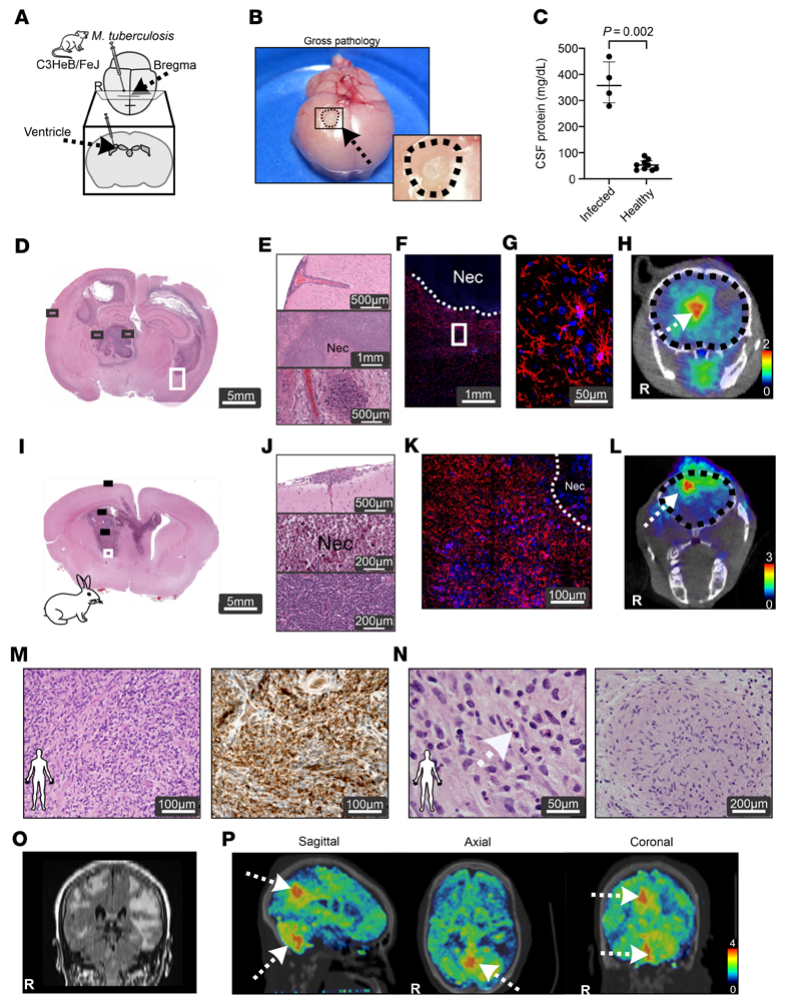
Animal models of TB meningitis recapitulate human disease. Mice (**A**–**H**), rabbits (**I**–**L**), and humans (**M**–**P**). (**A**) Schematic of brain infection of mice with live *M*. *tuberculosis*. Two weeks after infection, brain lesions were noted on gross pathology (**B**) with high protein in the CSF (*n* = 4–9 animals/group) (**C**). Histopathology from the brains of infected mice (**D**–**G**) and rabbits (**I**–**K**) demonstrates TB lesions with inflammatory cells. Panels **E** and **J** show meningitis (upper panels), necrotizing tuberculomas (middle panels), and nonnecrotizing tuberculomas (lower panels). Immunohistochemistry demonstrates microglia (Iba-1 stain in red and DAPI nuclear stain in blue) in brains from infected mice (**F** and **G**) and rabbits (**K**). ^18^F-FDG uptake is noted in the brain lesions on PET/CT images (arrows) from infected mice (**H**) and rabbits (**L**). Areas of nonspecific PET uptake were also noted extracranially. (**M** and **N**) Histology from brain lesion biopsies from 2 patients with TB meningitis (subjects 3 and 5, Supplemental Table 1) CD68^+^ cells (right, panel [**M**]) and multinucleated giant cells (left panel [**N**], white arrow). (**O**) MRI from a 67-year-old female with TB meningitis (subject 3, Supplemental Table 1) demonstrating focal FLAIR hyperintensities and ^18^F-FDG uptake (arrows) noted on PET/CT images (**P**). Coronal PET images are presented as standardized uptake values (SUV). High-power views (**D**, **E**, **I**, **J**, **M**, and **N**) are shown in Supplemental Figures 1 and 3, respectively. Data are represented as median ± IQR. Statistical comparisons were performed using a 2-tailed Mann-Whitney-Wilcoxon test (**B**). R, right; Nec, necrotizing.

C3HeB/FeJ female mice or New Zealand white rabbits were infected using direct intracranial inoculation with *M*. *tuberculosis* (Figure 2A). Exudative meningitis led to adhesion of the brain parenchyma to the skull and exudate at the site of injection, with preserved brain morphology (Figure 2B). Similarly to that of patients with TB meningitis, the CSF from the infected mice and rabbits (12) was noted to have significantly higher protein levels (median, 357.6; IQR, 291.1–468.2 mg/dL; *P* = 0.002) compared with that of healthy controls (Figure 2C). Histopathological analysis of brain tissues from *M*. *tuberculosis*–infected mice and rabbits revealed meningitis in combination with necrotizing and nonnecrotizing parenchymal lesions and activated microglia morphology (Figure 2, D–G and I–K, and Supplemental Figure 1; supplemental material available online with this article; https://doi.org/10.1172/JCI155851DS1). Inflammatory cells were also detected in the choroid plexus, lateral and fourth ventricles, with scattered multinucleated giant cells and foamy macrophages (Supplemental Figure 2). Focal ^18^F-FDG uptake at the infection sites in animals was noted on PET/CT imaging (Figure 2, H and L). Similar findings were noted in a patient with TB meningitis who underwent brain biopsy for clinical reasons, demonstrating parenchymal lesions with multinucleated giant cells and CD68^+^ cells (a marker of microglia/macrophages) (Figure 2, M and N, and Supplemental Figure 3). Focal fluid-attenuated inversion recovery (FLAIR) hyperintensities on MRI (Figure 2O) correlated with high ^18^F-FDG PET uptake (Figure 2P and Supplemental Figure 4) in patients with TB meningitis.

### High-dose rifampin increases bactericidal activity without increased intracerebral inflammation

Cross-species studies to evaluate a high-dose (35 mg/kg/d) rifampin-containing regimen in mice and rabbits with experimentally induced TB meningitis were performed (20, 21).

#### Mouse studies.

**Figure 3:**
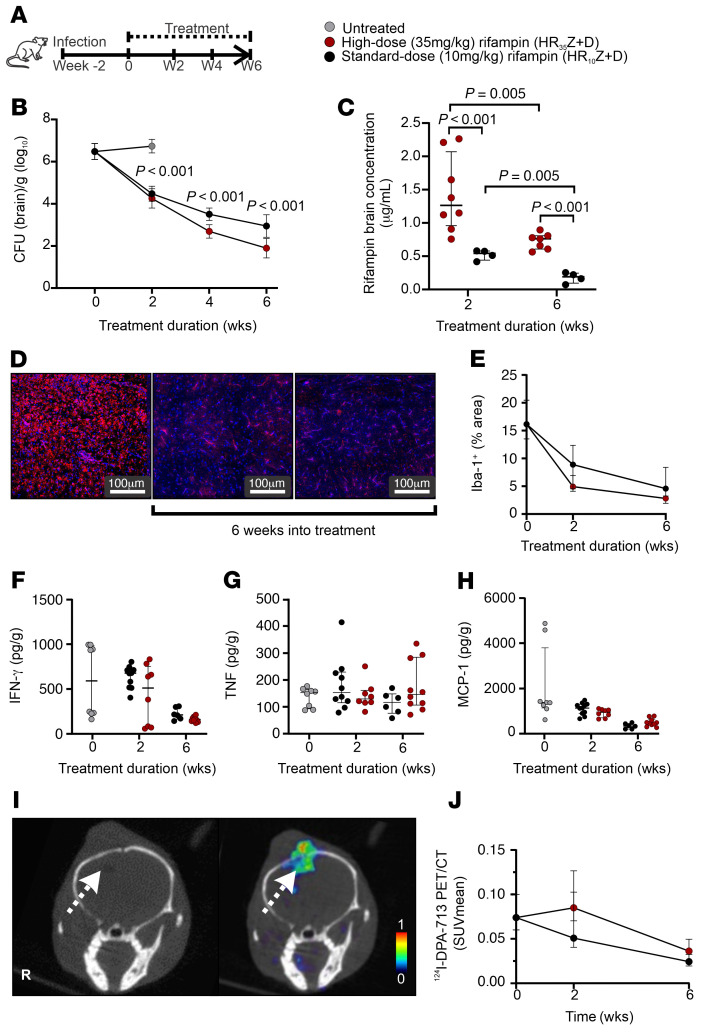
Treatment with a high-dose rifampin-containing regimen in mice. (**A**) Experimental schematic for multidrug treatment in mice with experimentally induced TB meningitis. Week –2 represents 2 weeks prior to initiation of TB treatments. (**B**) Bacterial burden (CFU per gram of brain tissue [log_10_]) (*n* = 9–16 animals/group per time point). (**C**) Rifampin brain concentration (μg/mL) (*n* = 4–8 animals/group). (**D**) Representative images from untreated (left panel) animals or animals treated with 6 weeks of high-dose (middle panel) or standard-dose (right panel) rifampin-containing regimen demonstrating microglia (Iba-1 stain in red and DAPI nuclear stain in blue) density in brain tissues. (**E**) Quantification of Iba-1 signal (*n* = 3 animals/group per time point). (**F**–**H**) Brain tissue levels of IFN-γ (**F**), TNF (**G**), and MCP-1 (**H**) (*n* = 3–6 animals/group per time point, with 2 technical replicates per animal). (**I**) ^124^I -DPA-713 PET/CT images from a representative mouse demonstrating a hypodense lesion (left, white arrow) on CT corresponding to ^124^I-DPA-713 PET activity (right, white arrow) at the site of a TB lesion. (**J**) Serial ^124^I-DPA-713 PET imaging presented as SUV_mean_ (*n* = 5–19 animals/group per time point). Data are represented as median ± IQR range except bacterial burden (CFU), which is presented as mean ± SD. Statistical comparisons were performed using 2-way ANOVA followed by Bonferroni’s multiple-comparison test (**B** and **C**).

Mice with experimentally induced TB meningitis were randomly allocated to receive a high-dose (human equipotent dose of 35 mg/kg/d) or standard-dose (human equipotent dose of 10 mg/kg/d) rifampin-containing TB regimen in combination with isoniazid, pyrazinamide, and dexamethasone (Figure 3A) at human equipotent doses. The high-dose rifampin-containing TB regimen demonstrated significantly higher bactericidal activity in the brain tissues at all time points and as early as 2 weeks after initiation of treatment (*P* < 0.001) (Figure 3B). At 6 weeks after initiation of treatment, brains of mice treated with the high- versus standard-dose rifampin-containing TB regimen had approximately 10-fold lower bacterial burden. Reductions in bacterial burden were also noted in the spleen of these mice (Supplemental Figure 5A; *P* = 0.02). At 2 weeks after treatment initiation, weight gain was observed in all animals, with significantly higher weight gain observed in mice treated with the high- versus the standard-dose rifampin regimen (Supplemental Figure 5B; *P* = 0.02).

Brain rifampin concentrations significantly decreased during TB treatment in both groups (Figure 3C; *P* = 0.005) and were consistent with our prior data (12). However, brain rifampin concentrations in mice receiving the high- versus standard-dose rifampin regimen were significantly higher at 2 and 6 weeks after initiating TB treatment (Figure 3C; *P* < 0.001). Rifampin plasma levels were also significantly higher in mice receiving the high- versus standard-dose rifampin regimen (Supplemental Figure 6A), and the rifampin brain/plasma ratios were no different between treatment regimens (Supplemental Figure 6B). Rifampin levels in the CSF were lower than in the brain tissue and undetectable in all mice treated with the standard-dose rifampin regimen, but detectable in 71% of mice treated with high-dose rifampin (Supplemental Figure 6C).

We also assessed intracerebral inflammation associated with the different treatment regimens. No significant differences were noted in microglia density (Iba-1^+^ staining) (Figure 3, D and E, and Supplemental Figure 7) or cytokine levels of IFN-γ, TNF, MCP-1 (or CCL-2), IL-10, and IL-17 (Figure 3, F–H, and Supplemental Figure 8, A and B) in brain tissue obtained from mice treated with the high- or standard-dose rifampin regimen. IL-6 levels were significantly lower in brain tissues obtained from mice treated with the high- versus standard-dose rifampin regimen 2 weeks after initiation of TB treatment (*P* = 0.001), but these differences were not evident at 6 weeks (Supplemental Figure 8C). Similarly, no differences were noted in IFN-γ, TNF, MCP-1 (or CCL-2), and IL-6 levels in CSF obtained from mice treated with the high- or standard-dose rifampin regimen (Supplemental Figure 9). Interestingly, cytokine levels were generally much higher in the CSF than in the brain tissues. Additionally, levels of certain cytokines (TNF, MCP-1) decreased substantially in the CSF by 2 weeks of treatment, but this was not evident in the brain tissues. Longitudinal imaging with ^124^I-DPA-713 PET was also performed to measure lesion-specific, intraparenchymal brain inflammation. While the ^124^I-DPA-713 PET signal decreased with treatment (19), no differences were noted in mice treated with the high- or standard-dose rifampin regimen (Figure 3, I and J).

#### Rabbit studies.

**Figure 4:**
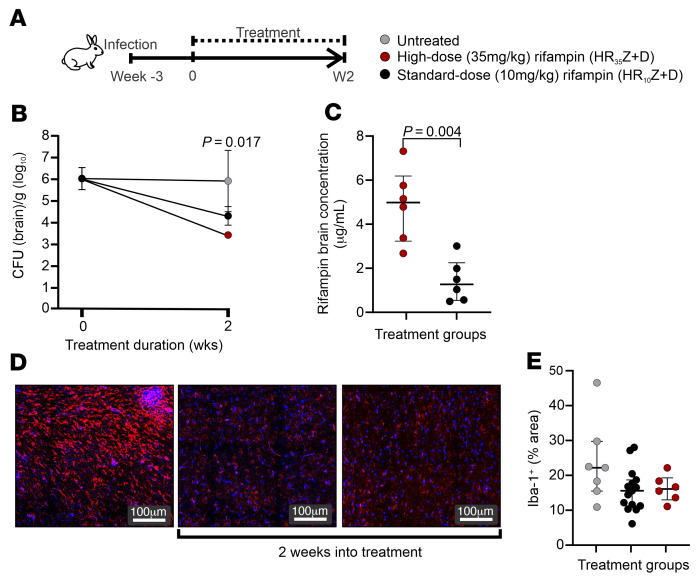
Treatment with a high-dose rifampin-containing regimen in rabbits. (**A**) Experimental schematic for multidrug treatment in New Zealand white rabbits with experimentally induced TB meningitis. Week –3 represents 3 weeks prior to initiation of TB treatments. (**B**) Bacterial burden (CFU per gram of brain tissue [log_10_]) (*n* = 3–4 animals/group per time point). (**C**) Rifampin brain concentration (μg/mL) (*n* = 3–4 animals/group with 1–2 samples/animal). (**D**) Representative images from untreated (left panel) rabbits or rabbits treated with 2 weeks of high-dose (middle panel) or standard-dose (right panel) rifampin-containing regimen demonstrating microglia (Iba-1 stain in red and DAPI nuclear stain in blue) density in brain tissues. (**E**) Quantification of Iba-1 signal (*n* = 1 animal/group). Data are represented as median ± IQR except for bacterial burden (CFU), which is presented as mean ± SD. Statistical comparisons were performed using 2-way ANOVA followed by Bonferroni’s multiple-comparison test (**B**) and 2-tailed Mann-Whitney-Wilcoxon test (**C**).

Male and female rabbits with experimentally induced TB meningitis were similarly randomly allocated to receive the high- (human equipotent dose of 35 mg/kg/d) or standard-dose (human equipotent dose of 10 mg/kg/d) rifampin-containing TB regimen in combination with isoniazid, pyrazinamide, and dexamethasone (Figure 4A). Consistent with the data obtained from mice, the high-dose rifampin-containing TB regimen demonstrated significantly higher bactericidal activity (~10-fold) in the brain tissues at 2 weeks after initiation of TB treatment (Figure 4B; *P* = 0.017). As expected, reductions in bacterial burden were also noted in the spleen (Supplemental Figure 10A). Rabbits treated with the high- versus the standard-dose rifampin regimen gained more weight, but these results were not statistically significant (Supplemental Figure 10B). Brain rifampin concentrations in rabbits receiving the high- versus standard-dose rifampin regimen were significantly higher (Figure 4C; *P* = 0.004). Rifampin levels in the plasma and the CSF were higher in rabbits treated with the high- versus the standard-dose rifampin regimen, though this was not statistically significant (Supplemental Figure 11), but CSF levels were lower than in the brain tissue. Finally, no significant differences were noted in microglia density (Iba-1^+^ staining) (Figure 4, D and E) in brain tissue obtained from rabbits treated with the high- or standard-dose rifampin regimen, which reproduced the findings in the mouse model.

### Dexamethasone affects rifampin exposure, bactericidal activity, and intracerebral inflammation

We studied the effect of dexamethasone, which is the standard of care for the treatment of TB meningitis, on rifampin-containing regimens in mice. Compared with regimens without dexamethasone, the bactericidal activity was lower for dexamethasone-containing standard- and high-dose rifampin regimens (Supplemental Figure 12). Brain rifampin concentration and rifampin brain/plasma ratios were lower in mice receiving regimens with dexamethasone (Supplemental Figure 12, C and D; *P* = 0.024). However, intracerebral inflammation measured by ^124^I-DPA-713 PET was also lower in mice receiving dexamethasone compared with those who were not (Supplemental Figure 12E; *P* = 0.006). Similarly, brain-tissue IFN-γ and IL-6 levels were lower in mice receiving dexamethasone (Supplemental Figure 12, F–K; *P* < 0.001 at 2 weeks [IFN-γ and IL-6] and *P* < 0.044 at 6 weeks [IFN-γ]).

### Localized blood-brain barrier disruption is an important driver of rifampin brain exposure

#### Animal studies.

**Figure 5:**
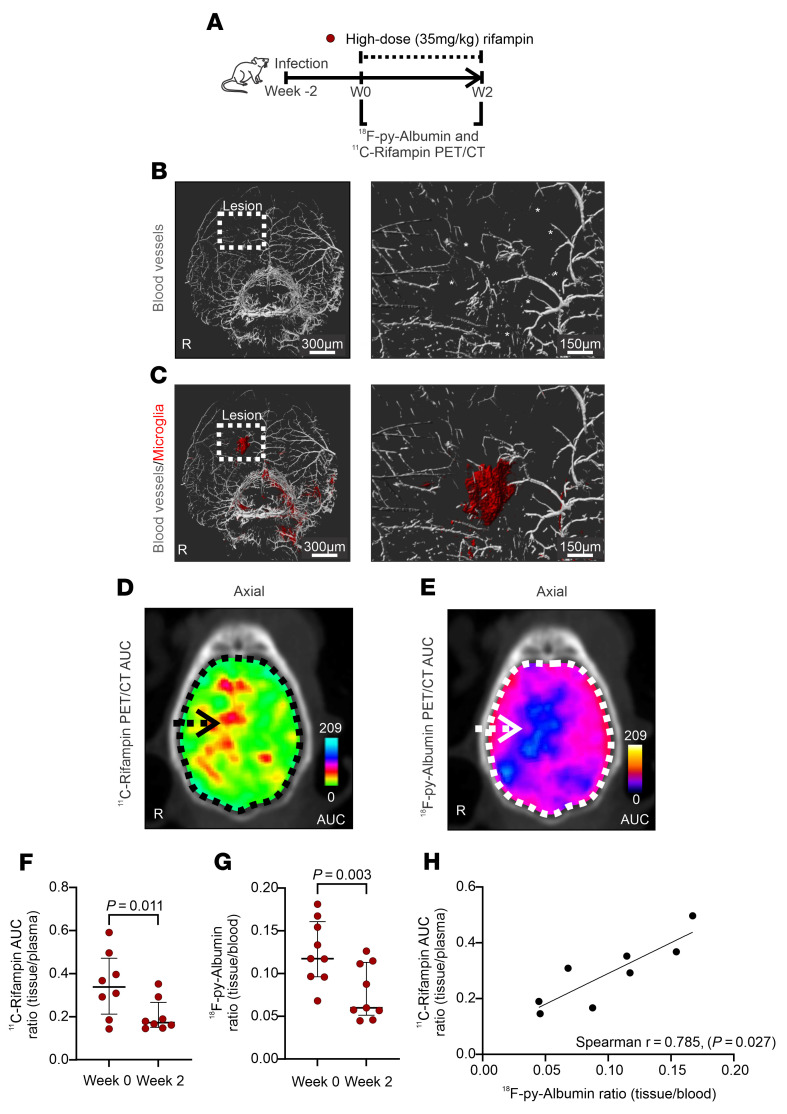
Spatiotemporal changes in rifampin brain exposures and vascular pathology in mice. (**A**) Experimental schematic. Whole brain immunostaining (iDISCO) was performed to visualize arteries (**B**, α-SMA stain in white) and microglia (**C**, Iba-1 in red) in infected mice. Truncated arteries are marked with asterisks. (**D**) ^11^C-Rifampin PET AUC shown as a heatmap overlaid on the axial CT section. (**E**) Corresponding ^18^F-py-Albumin PET AUC heatmap. Arrows point to the lesion, while the dotted line outlines the brain parenchyma. (**F**) ^11^C-Rifampin brain/plasma AUC ratios (*n* = 4 animals per time point). (**G**) ^18^F-py-Albumin brain/plasma ratio (*n* = 9 animals per time point). (**H**) Correlation between the lesion-specific ^18^F-py-Albumin PET uptake and ^11^C-rifampin exposures. Data are represented as median ± IQR. Statistical comparisons were performed using 2-tailed Mann-Whitney-Wilcoxon test (**F** and **G**) and Spearman’s rank correlation (**H**).

Rifampin has limited penetration into the CNS, which also decreases rapidly as early as 2 weeks after treatment initiation (12, 22). Therefore, we performed studies to explore the mechanisms underlying the spatiotemporal changes in rifampin brain exposures with the goal of identifying mechanisms that could be modulated to optimize rifampin delivery to the CNS. Immunostaining of clarified whole mouse brains with experimentally induced TB meningitis was performed to visualize in 3D, blood vessel pathology at high resolution (1 μm). These studies demonstrated vascular pathology consistent with interrupted blood supply (Figure 5, A and B, and Supplemental Figure 13) associated with localized areas of increased microglia density in TB lesions (Figure 5C and Supplemental Video 1). We also tested to determine whether changes in localized blood-brain barrier disruption could be driving the changes in rifampin brain exposure. Studies with ^18^F-py-Albumin, an imaging biomarker of blood-brain barrier disruption (23) (Supplemental Figure 14), and dynamic ^11^C-rifampin PET acquisitions were performed sequentially in the same set of mice. Dynamic imaging allowed measurements of time-concentration profiles rather than the single time-point measurements possible with direct tissue measurements in postmortem tissue samples. Spatially compartmentalized ^11^C-rifampin brain exposures (Figure 5D) were noted to colocalize with the ^18^F-py-albumin PET signal (Figure 5E). Both ^11^C-rifampin exposures (Figure 5F) and the ^18^F-py-albumin PET signals (Figure 5G and Supplemental Figure 14E) decreased significantly by 2 weeks after initiation of TB treatment (*P* < 0.03). Additionally, there was a significant correlation between the lesion-specific ^18^F-py-albumin PET uptake and ^11^C-rifampin exposures (Figure 5H; *r* = 0.785, *P* = 0.027). Finally, we evaluated the expression of MDR-1, a P-glycoprotein efflux pump, which can decrease CNS rifampin levels (24). While TB treatment (with rifampin-based regimens) increased MDR-1 expression, there was no significant difference in brain tissues obtained from mice treated with the high- or standard-dose rifampin regimen (Supplemental Figure 15).

#### Imaging studies in patients with TB meningitis.

**Figure 6:**
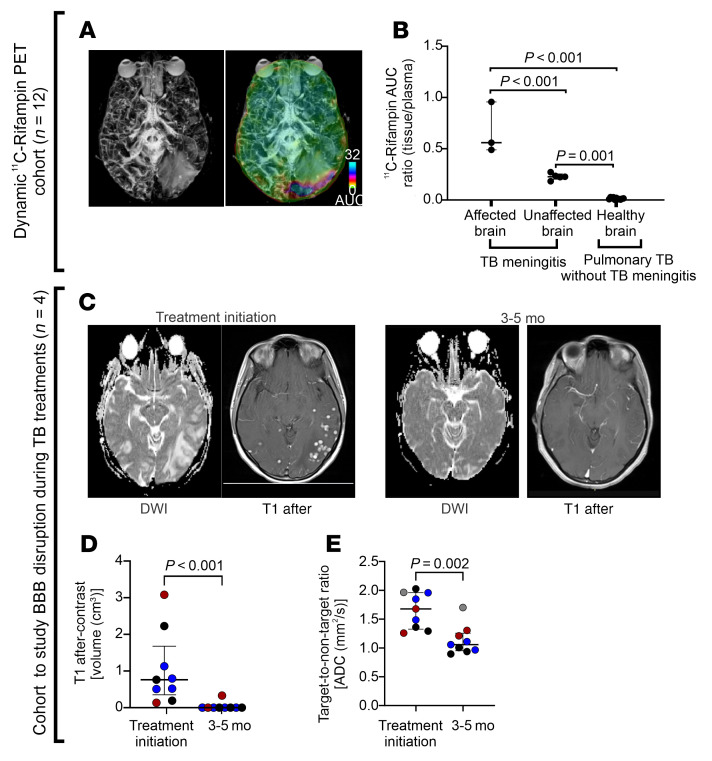
Imaging studies in patients with TB meningitis. (**A** and **B**) ^11^C-Rifampin PET/CT to study rifampin exposures. (**A**) MRI T2 FLAIR maximum intensity projection (MIP) (right) with the corresponding ^11^C-rifampin PET AUC overlaid as a heatmap (left). (**B**) ^11^C-Rifampin brain/plasma AUC ratios in brain regions with and without vasogenic edema in a patient with TB meningitis (*n* = 1 patient with 7 VOI) and in brains of patients with pulmonary TB, but without meningitis (*n* = 11 patients, 1 VOI per patient). (**C**–**E**) Another cohort of patients with TB meningitis who underwent serial MRI during TB treatment was used to assess the blood-brain barrier disruption (*n* = 4 patients). (**C**) Representative MRI axial sections with DWI and T1 after contrast at treatment initiation (left) and after 3 months of treatment (subject 1, Supplemental Table 1). (**D**) Changes in brain T1 after contrast volume (cm^3^) during TB treatment (*n* = 3; no contrast was administered for subject 4 with chronic renal disease). (**E**) Changes in brain diffusion (ADC [mm^2^/s]) for all 4 patients. All patients received 2 months of initiation treatment with HRZ with or without fluoroquinolones, followed by continuation phase with at least 12 months of HR treatment. Panel **C** and the corresponding T2 FLAIR are shown in Supplemental Figure 18A. Data are represented as median ± IQR. Statistical comparisons were performed using 2-way ANOVA followed by Bonferroni’s multiple-comparison test (**B**) and 2-tailed Mann-Whitney-Wilcoxon test (**D** and **E**). TB drug treatments are abbreviated. BBB, blood-brain barrier; T1 post, T1 after contrast image.

Imaging studies in 2 cohorts of patients with TB meningitis were used to validate the animal data regarding spatiotemporal changes in rifampin exposures. Brain rifampin exposures in 12 newly identified patients with TB enrolled through a first-in-human study utilizing dynamic ^11^C-rifampin PET (10, 12) were analyzed. Consistent with the animal data, rifampin brain exposures were significantly higher in a patient with TB meningitis versus patients with pulmonary TB (*n* = 11), but without meningitis (Figure 6, A and B; *P* < 0.001). Additionally, and also consistent with the animal data, ^11^C-rifampin brain exposures were significantly higher in regions with vasogenic edema noted on MRI (vascular leak at the site of TB lesions) versus regions without edema (Figure 6, A and B; *P* < 0.001).

Medical records at the Johns Hopkins Hospitals were queried to identify patients with confirmed TB meningitis who also underwent serial MRI during TB treatment (Figure 6, C–E, and Supplemental Figure 16, and Supplemental Table 1). Blood-brain barrier disruption was estimated using volumetric measurements (cm^3^) of enhancing brain tissues on postcontrast T1-weighted scans (sites of leakage of gadolinium-based contrast agents [GBCA]), demonstrating a significant decrease at 3 to 5 months after initiation of TB treatment (Figure 6D; *P* < 0.001; Supplemental Figure 18). Apparent diffusion coefficient (ADC), which is an indirect measure of brain edema in this setting, also demonstrated a significant decrease in all patients at 3 to 5 months after initiation of TB treatment (*P* = 0.002) (Figure 6E and Supplemental Figure 18). One patient (subject 3, Supplemental Table 1) developed paradoxical worsening (new brain lesion) after initiation of TB treatment, which resolved with continued treatment (Supplemental Figure 17).

## Discussion

Animal models have played an essential role in drug development for infectious diseases, especially TB (25). However, unlike with pulmonary TB, animal models have not been widely utilized to evaluate new TB drugs for TB meningitis (2). To date, several mouse strains have been used to recapitulate TB meningitis (2, 26–28), but these suffer from variability. Here, we describe a tractable murine model of TB meningitis in C3HeB/FeJ mice using direct intraventricular inoculation, which mimics key features of TB meningitis in humans.

Cross-species studies were performed to evaluate a high-dose (35 mg/kg/d) rifampin-containing regimen in mice and rabbits with experimentally induced TB meningitis demonstrating that high-dose rifampin leads to faster bacterial killing as early as 2 weeks after treatment initiation, which is likely due to the observed increased drug exposure in the brain. While all drugs were administered orally for the duration of the TB treatments in both mice and rabbits, rifampin levels were obtained at 30 minutes after an intravenous dose in rabbits and 4 hours after an oral dose in mice corresponding to the time to reach plasma C_max_(T_max_) in both species. Therefore, higher rifampin levels were noted in rabbits versus mice. Importantly, these rifampin levels are consistent with studies evaluating CSF rifampin levels in adults (29) and children (30) with TB meningitis.

It has been hypothesized that outcomes in TB meningitis may be more strongly associated with changes in intracerebral inflammation rather than bacterial killing (15, 31). This is relevant, as intensified TB regimens with enhanced bacterial killing could worsen intracerebral inflammation due to the release of proinflammatory components during bacterial lysis (32). However, rifampin has been shown to release fewer bacterial proinflammatory products than other antibiotics (e.g., β-lactams) (31, 32), and one study demonstrated that it also reduces early mortality in experimental *Streptococcus pneumoniae* meningitis in mice (33), although there may be differences in pathogenesis of meningitis due to *S. pneumoniae* and *M*. *tuberculosis*. CT and ^18^F-FDG PET are being increasingly used to monitor TB treatment. However, both technologies lack specificity for TB-associated inflammation. Here, noninvasive imaging was utilized to serially monitor the same cohort of mice using ^124^I-DPA-713 PET, a clinically translatable imaging biomarker for TB-associated inflammation (specific for activated microglia/macrophages) (16–19), which demonstrated no differences in lesion-specific, intraparenchymal brain inflammation in animals treated with either high- or standard-dose rifampin-containing regimen. Postmortem analyses of brain tissues and CSF were consistent with the imaging data. Additionally, cytokine levels in the CSF of mice declined with TB treatment, which is consistent with the trends noted in cytokine levels in lumbar and ventricular CSF obtained from patients with TB meningitis (34, 35). Overall, the cytokine levels in the CSF did not correlate with those noted in the brain tissues. Similarly, rifampin levels in the CSF were also discordant with those in the brain parenchyma and consistent with our prior findings (12), reinforcing that drug concentrations, inflammation, and likely bacterial burden are spatially compartmentalized. This is an important finding, as CSF studies are commonly utilized in many clinical trials, but CSF may not be an adequate surrogate of disease in TB meningitis. Due to the limited availability of species-specific reagents, cytokine analyses were not feasible in rabbit tissues. However, consistent with the mouse data, no significant differences were noted in microglia density in brain tissues obtained from rabbits treated with the high- or standard-dose rifampin regimen.

Dexamethasone is included in the standard of care for the treatment of patients with TB meningitis (36, 37). However, the benefit of steroids in HIV-positive patients with TB meningitis remains unclear. Additionally, genetic polymorphisms that modulate inflammatory responses in the host, e.g., leukotriene A4 hydrolase expression (LTA4H), tryptophan metabolism (36), may affect mortality in patients with TB meningitis. For example, TT homozygotes, with increased expression of LTA4H, had the highest survival benefit when treated with dexamethasone in a Vietnamese cohort, although this was not observed in an Indonesian cohort (37). Indeed, steroids may only confer a modest survival benefit in studies, likely secondary to the absence of the T allele in the vast majority of people worldwide (37). Conversely, dexamethasone is associated with a number of adverse events (38, 39) and has drug-drug interactions with rifampin (40). Here, we demonstrate that adjunctive use of dexamethasone in mice with experimentally induced TB meningitis results in lower bactericidal activity. This was associated with reduced intraparenchymal rifampin levels, likely due to healing of the blood-brain barrier disruption by corticosteroids. Nevertheless, the beneficial antiinflammatory effects of dexamethasone were shown in the brain by the reduction of inflammation biomarkers (e.g., IFN-γ and IL-6). While identified originally as a B cell differentiation factor, IL-6 plays a key role in the physiological homeostasis and pathogenesis of neuroinflammation (41). Interestingly, dexamethasone does not alter the CSF proinflammatory cytokines or chemokines in patients with TB meningitis (38), further highlighting that disease pathology in TB meningitis is compartmentalized.

The CNS is separated from the systemic circulation by the blood-brain barrier and the blood-CSF barrier (together often referred to as the blood-brain barrier), which limit penetration of exogenous substances into the brain parenchyma and the CSF compartments, respectively. Additionally, the presence of cerebral stroke, vasculitis, and vasospasm increases the risk of brain hypoxia and drive host immune response (42–44). Whole-brain immunostaining of clarified mouse brains with experimentally induced TB meningitis demonstrated arterial damage and inflammation at the site of TB lesions. Multimodal imaging in animals revealed spatially compartmentalized brain rifampin exposures with increased exposures in regions with blood-brain barrier disruption. Consistent with the animal data, brain rifampin exposures in patients imaged with dynamic ^11^C-rifampin PET/CT (10, 12, 30, 45) were spatially compartmentalized with substantially higher rifampin exposures in regions with vascular leak and brain edema. We have previously demonstrated significant decreases in rifampin brain exposures in the rabbit model using ^11^C-rifampin PET/CT as early as 2 weeks after initiation of TB treatment (12). The data from the mouse model not only corroborate these results, but also provided a possible mechanistic explanation by demonstrating decreased blood-brain barrier disruption at 2 weeks of treatment as well. The second cohort of 4 patients with TB meningitis demonstrated a significant (*P* <0.001) decrease in the lesion-specific blood-brain barrier disruptions at 3 to 5 months after initiation of TB treatment, confirming the blood-brain barrier healing seen in the mouse model. Finally, while TB treatment (with rifampin-based regimens) modestly increased MDR-1 expression, there was no significant difference in MDR-1 expression in brain tissues obtained from mice treated with the high- or standard-dose rifampin regimen. However, additional studies are needed to assess the role of MDR-1 (or other mechanisms), which could be modulated to enhance rifampin exposure in the CNS.

Our studies have some limitations. While treatment for TB meningitis is for 12 months, we performed studies at early time points (up to 6 weeks). This is because most deaths (and neurological damage) in TB meningitis occur early (13, 45), with one study reporting the majority of deaths within 2 weeks of hospital admission (43, 46), highlighting the need for early interventions for TB meningitis (47). The animal models utilized in this study used direct intracranial inoculation with *M*. *tuberculosis*, which, while not the natural route of infection (47), recapitulated key pathological features of human TB meningitis. While both male and female rabbits were utilized in the current studies, only female mice were utilized and additional studies with both male and female mice are required to understand sex differences in TB meningitis. Additionally, collection of CSF in mice remains technically challenging, and therefore analyses were limited to a single time point (2 weeks) after initiation of TB treatment. Although we provide biological surrogates for efficacy (bactericidal activity and neuroinflammation), we plan to include additional clinical readouts (e.g., time to death) in future studies. Microdoses (nanograms to micrograms) of ^11^C-rifampin were administered per subject for the PET studies (48). Direct measures of rifampin tissue levels were performed at least 2 weeks after initiation of a rifampin-based TB treatment in animals when autoinduction of hepatic metabolism had peaked (1, 10). Similarly, all TB patients undergoing ^11^C-rifampin PET had received at least 10 days of a rifampin-based regimen at the time of the studies (10). While ^11^C-rifampin PET data on brain penetration was obtained from all the TB patients, only one patient had TB meningitis. Finally, despite the lack of temporal sequential ^11^C-rifampin PET, the MRI data in patients with TB meningitis support that blood-brain barrier disruption is lesion specific and that this disruption in the blood-brain barrier function significantly (*P* < 0.001) decreases after initiation of TB treatment.

Clinical trials are expensive and can take many years to complete, especially for TB meningitis, which is a less common form of TB. Additionally, patients in different disease stages (early and advanced) are enrolled in the same clinical trials, which has become an important confounder for evaluating new treatments (2). Our data suggest that animal models of TB meningitis can be used to evaluate and prioritize promising treatments prior to their evaluation in clinical trials. State-of-the-art, clinically translatable imaging approaches can also expedite unbiased, cross-species studies and validation of animal data in relevant human populations (10, 49, 50). Finally, while rifampin is a key first-line TB drug, it is widely used to treat serious infections due to other pathogens, e.g., *Staphylococcus aureus* (and methicillin-resistant *S*. *aureus* [MRSA]) (51). Therefore, our studies are broadly applicable to other infections and could enable personalized medicine in resource-rich settings.

## Methods

### Animal studies.

Female C3HeB/FeJ mice (7 to 8 weeks old, Jackson Laboratories) were infected through a burr hole (Micro-Drill Kit, Braintree Scientific Inc.) using a stereotaxic instrument (David KOPF Instruments, model 900) at specified coordinates (0.6 mm dorsal to bregma, 1.2 mm lateral to middle line, and 2 mm ventral) for intraventricular injection. Titrated frozen stocks were used to implant 6.41 ± 0.08 log_10_ CFU of *M*. *tuberculosis* H37Rv in 2.7 μL or PBS using a 5 μL Hamilton syringe (Hamilton, 88000). CSF was obtained from mice using a published protocol (52) modified for use in an animal biosafety level-3 (ABSL-3) facility. Protein levels were measured using a BCA Protein Assay Kit (Thermo Fisher). Titrated frozen stocks were used to implant 5.57 ± 1.09 log_10_ CFU in male and female New Zealand white rabbits (5 to 6 days old, Robinson Services Inc.), as described previously (16), but modified for intraventricular injection. Based on human equivalent neurodevelopment at the time of infection, the mouse and rabbit models represent adult and pediatric (6 to 12 months old) age groups, respectively (20, 53). Treatments began 2 and 3 weeks after infection in mice and rabbits, respectively.

Experimentally infected mice and rabbits were randomly allocated to receive standard-dose (10 mg/kg/d) or high-dose (35 mg/kg/d) rifampin (R_10_ and R_35_, respectively) in combination with isoniazid, pyrazinamide, and dexamethasone by oral gavage (except that dexamethasone was administered intraperitoneally in mice) 5 days per week at human equipotent doses (Supplemental Table 2) (54, 55). Dexamethasone dose was based on the already known biological effect in mice (56, 57) and dosing conversion between mice and humans (55) as well as rabbits and humans (54). In mice, rifampin dose prior to sample collection for mass spectrometry was administered by oral gavage and tissue harvested at the time to reach plasma C_max_(T_max_) (58). However, due to the unique digestive physiology in rabbits, on the day of sacrifice, rifampin was administered intravenously with sample collection performed 30 minutes after the dose, consistent with our previous studies (10, 12). Quantification of bacterial burden in whole-brain and spleen tissues was performed as described previously (10). Multiplex Luminex assays (EMD Millipore) were performed to measure cytokine levels and normalized to protein. Evans blue extravasation assays were performed in *M*. *tuberculosis*–infected and control (PBS injected) animals injected intravenously with 5 mL/kg of 2% (w/v) Evans blue. One hour after injection, animals were sacrificed and perfused with PBS through the heart (left ventricle). Evans blue was visualized and quantified by optical imaging (excitation at 620 nm, emission at 680 nm) using the IVIS Lumina LT (PerkinElmer).

Tissues were incubated overnight at 4°C with primary antibodies targeting Iba-1 (Wako, 019-19741, 1:500) and P-glycoprotein (Novus Biological, catalog 1007906, 1:100) for mice and Iba-1 (Abcam, catalog ab107159, 1:500) for rabbit tissues. Secondary goat (Thermo Fisher, catalog A11011) or donkey (Thermo Fisher, catalog A32849) Alexa Fluor antibodies were utilized and imaged with an Nikon A1^+^ confocal microscope. Data analyses were performed using HALO (Indica Labs). To decrease bias, regions of interest were drawn at the edge of TB lesions to quantify immunofluorescence in predefined zones A (<500 μm), B (>500 μm to <1,000 μm), and C (>1,000 μm); measurements indicate distance from border of the lesion (Supplemental Figure 7). All zones had the same area (0.26 mm^2^). Immunostaining of clarified whole mouse brains was performed to visualize pathology in 3D using the iDISCO protocol (59). Vasculature and microglia were labeled by targeting α-SMA (Abcam, 1000093) and Iba-1 (Wako, 1037485), respectively. Whole brains were imaged with light sheet microscopy (LaVision BioTec UltraMicroscope II) and visualized and quantified using Imaris (version 9.2). Same-size volumes of interest (VOIs) were created on the lesion and contralateral areas. Quantification of α-SMA intensity was represented as percentage of area.

Plasma, CSF, and brain tissues were assayed using validated ultra-high-performance liquid chromatography (UPLC) and liquid chromatography–tandem mass spectrometry (LC–MS/MS) for rifampin at the Infectious Diseases Pharmacokinetics Laboratory of the University of Florida (standard curves from 50.00 to 0.05 μg/mL). The assays measured both the free and protein-bound rifampin. Calibration curves were prepared in plasma and in artificial CSF, and assay performance was similar in both. The 25-desacetyl rifampin was not measured in mice and was undetectable in rabbit CSF and brain tissues.

### Human studies.

Studies were performed in 2 cohorts of TB patients. The first cohort comprised 12 TB patients enrolled from January 2017 to February 2019 in a first-in-human study utilizing dynamic ^11^C-rifampin PET/CT (10) at the Johns Hopkins Hospitals. All patients received at least 10 days of TB treatment by the time of imaging. The second cohort of 4 patients with TB meningitis was established by retrospective analysis of medical records at the Johns Hopkins Hospitals from July 2011 to July 2021 to identify patients with confirmed drug-susceptible (microbiology or molecular methods) TB meningitis who underwent longitudinal MRI assessments during TB treatment. Blood-brain barrier disruption was estimated using volumetric measurements (cm^3^) of enhancing brain tissues on postcontrast T1-weighted scans (sites of leakage of GBCA). ADC, which measures the diffusion magnitude of water molecules in tissues, was used as an indirect measure of brain edema. Brain tissue biopsies (performed for clinical reasons) were utilized for histopathological analysis.

### Imaging.

^11^C-Rifampin was synthesized at the Johns Hopkins PET Center using current good manufacturing practices. ^124^I-DPA-713 and ^18^F-py-albumin (methods adapted from refs. 60, 61) were synthesized for animal use. ^18^F-FDG was purchased from Sofie Co.

Live *M*. *tuberculosis*–infected animals were imaged inside transparent and sealed biocontainment cells compliant with BSL-3 containment and capable of delivering air-anesthetic mixture to sustain live animals during imaging, as previously described (19). PET/CT acquisition was performed on the nanoScan PET/CT (Mediso) after intravenous injection of the radiotracer as follows: 15 minutes static PET 45 minutes after injection of ^18^F-FDG (5.8 ± 0.3 MBq) after fasting for at least 8 hours; 20 minutes static PET 24 hours after injection of ^124^I-DPA-713 (8.6 ± 0.5 MBq); and dynamic PET for 40 minutes immediately after injection of ^11^C-rifampin (8.9 ± 5.1 MBq) or ^18^F-py–albumin (6.2 ± 0.4 MBq). In animals undergoing multimodal imaging, radiotracer injection was separated by 1 day and performed sequentially using from short to long half-life radiotracers (i.e., ^11^C first, ^18^F, and finally ^124^I) to avoid contamination from residual radiotracer. Images were reconstructed and coregistered using VivoQuant, version 3.5 (InviCRO), and spherical VOIs were drawn to measure PET activity or time-activity curves in the blood (left ventricle), brain lesions, and contralateral unaffected regions. Whole blood VOIs were corrected to plasma using the average hematocrit (45%) in mice. Heatmap overlays were implemented using RStudio Version 1.2.1335 (R Foundation).

Human studies were conducted as previously reported (10, 12). Briefly, a dynamic PET/CT (Biograph mCT, Siemens) was performed for 45 minutes (midabdomen to the skull vertex) immediately after an intravenous injection of ^11^C-rifampin (337 ± 14 MBq). Images were reconstructed and coregistered using PMOD (PMOD Technologies LLC). 3D spherical VOIs were drawn to measure ^11^C-rifampin in the blood (left ventricle), brain lesions (visualized with T1 postcontrast and T2 FLAIR MRI sequences), and contralateral unaffected brain regions. Whole blood VOIs were corrected to plasma using the patient hematocrit. PET/CT was performed after intravenous injection of ^18^F-FDG (310-318 MBq) on a Discovery DRX PET/CT scanner (GE Healthcare) or Biograph mCT PET/CT. The “hot contour ROI” PMOD tool was used to measure lesion volumes in T1 after contrast within the high-intensity areas. 3D spherical VOIs of the same size were drawn to measure the ADC on diffusion-weighted imaging (DWI). Due to the logistics of scanning patients, some MRI scans were acquired on different scanners. This does not affect quantification of T1 postcontrast enhancement, as volume rather than intensity of enhancing tissues was used for the current studies. Similarly, for ADC quantification, an internal normalization approach was utilized in which ADC values in the relevant involved VOIs were normalized to contralateral VOIs. This ratio-based method eliminates the variability of ADC measures across scanners.

### Statistics.

Prism 9.2 (GraphPad Software Inc.) was used. Data are represented as median ± IQR except bacterial burden (CFU), which is presented as mean ± SD. Multiple comparisons were performed using 2-way repeated-measures ANOVA followed by Bonferroni’s multiple-comparison test. Significance between 2 groups was determined with 2-tailed *t* test (parametric distribution) or Mann-Whitney-Wilcoxon test (nonparametric distribution). Correlation analysis for PET imaging was performed using Spearman’s correlation analysis. *P* ≤ 0.05 was considered statistically significant. Sample size, selection, and replicates are provided in the figure legends. Although animal and human studies were not blinded, a unique identification number was provided to each subject and measurements were made without knowledge of group assignment.

### Study approval.

All protocols were approved by the Johns Hopkins University Biosafety, Radiation Safety, Animal Care and Use, and IRB committees. The first cohort comprised 12 TB patients in a first-in-human study utilizing dynamic ^11^C-rifampin PET/CT (10, 12) at the Johns Hopkins Hospitals per the US FDA Radioactive Drug Research Committee program guidelines (62, 63). These studies were approved by the Johns Hopkins University IRB Committee and the Maryland Department of Health IRB. Written, informed consent was obtained from all participants, and there was no external data and safety monitoring board. The second cohort of 4 patients with TB meningitis was established by retrospective analysis of medical records at the Johns Hopkins Hospitals. Only deidentified images are presented.

## Author contributions

CARB and SKJ conceptualized and designed the studies. CARB, FM, MB, KF, PDJ, and AAO performed the mouse studies. EWT, CE, MB, KF, and JK performed the rabbit studies. EWT supervised the rabbit studies. CARB, FM, and MB analyzed the animal PET/CT data. FJM, CE, AAO, and CARB performed and analyzed the immunofluorescence data. FJM and CE performed the iDISCO studies. CARB and FJM analyzed the iDISCO data. FM and PDJ synthesized ^18^F-py-albumin. CAF synthesized ^124^I-DPA-713. CA Peloquin performed mass spectrometry for rifampin. MIRM and CA Pardo supervised the extraction of data from the cohort to study blood-brain barrier disruption during TB treatment. CARB and AAO analyzed the human PET/CT data. CARB and DAH analyzed the human MRI data. CARB and SKJ collated and analyzed all the data in this manuscript and performed statistical analyses. CARB, FM, EWT, and SKJ wrote the initial draft, and all coauthors edited the manuscript. SKJ obtained funding and supervised the project.

## Supplementary Material

Supplemental data

Supplemental video 1

## Version 1 01/27/2022

In-Press Preview

## Version 2 03/15/2022

Electronic publication
