# Supplementary material for: High-dose rifampin improves bactericidal activity without increased intracerebral inflammation in animal models of tuberculous meningitis
Source: J Clin Invest. 2022 Mar 15;132(6):e155851. doi: 10.1172/JCI155851 (PMC8920328; doi:10.1172/JCI155851)
Supplement: Supplemental data [file jci-132-155851-s091.pdf]

Supplementary Materials for

High-dose rifampin improves bactericidal activity without increased intracerebral inflammation in animal models of tuberculous meningitis

Authors: Camilo A. Ruiz-Bedoya[1,2,3*], Filipa Mota[1,2,3*], Elizabeth W. Tucker[1,2,4*], Farina J. Mahmud[1,2,3], Maria I. Reyes-Mantilla[5], Clara Erice[1,2,4], Melissa Bahr[1,2,3], Kelly Flavahan[1,2,3], Patricia de Jesus[1,2,3], John Kim[1,2,4], Catherine A. Foss[6], Charles A. Peloquin[7], Dima A. Hammoud[8], Alvaro A. Ordonez[1,2,3], Carlos A. Pardo[5,9], Sanjay K. Jain[1,2,3,6#]

Affiliations:

[1]Center for Infection and Inflammation Imaging Research, Johns Hopkins University School of Medicine, Baltimore, MD 21287, USA
[2]Center for Tuberculosis Research, Johns Hopkins University School of Medicine, Baltimore, MD 21287, USA
[3]Department of Pediatrics, Johns Hopkins University School of Medicine, Baltimore, MD 21287, USA
[4]Department of Anesthesiology and Critical Care Medicine, Johns Hopkins University School of Medicine, Baltimore, MD 21287, USA
[5]Department of Neurology, Johns Hopkins University School of Medicine, Baltimore, MD 21287, USA
[6]Russell H. Morgan Department of Radiology and Radiological Sciences, Johns Hopkins University School of Medicine, Baltimore, MD 21287, USA
[7]Infectious Disease Pharmacokinetics Laboratory, Pharmacotherapy and Translational Research, University of Florida College of Pharmacy, Gainesville, FL 32610, USA
[8]Center for Infectious Disease Imaging, Radiology and Imaging Sciences, National Institutes of Health, Bethesda, MD 20892, USA
[9]Department of Pathology, Johns Hopkins University School of Medicine, Baltimore, MD 21287, USA

*Authors contributed equally

[#]Corresponding author. Address: 1550 Orleans Street, Baltimore, MD 21287. Office Rm 1.09

Phone: 410-502-8241 Email: sjain5@jhmi.edu

Competing interests: All authors declare that they have no competing interests.

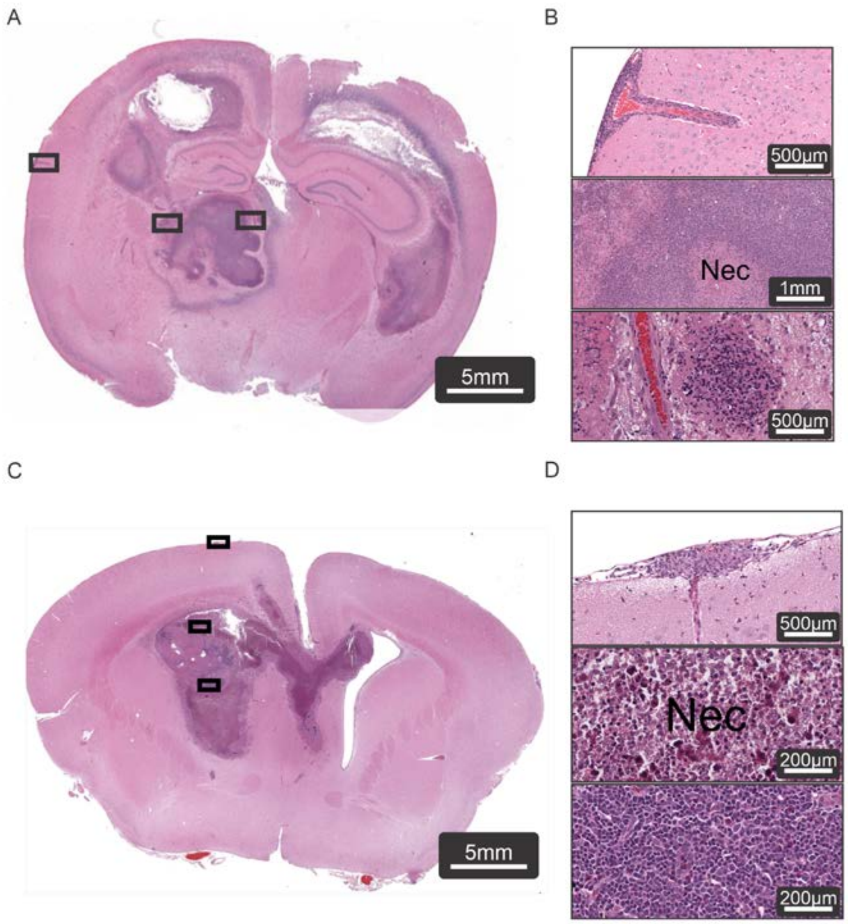

Supplemental Fig. 1. Histopathology in mice and rabbits with TB meningitis. Histopathology from the brains of infected mice (**A-B**) and rabbits (**C-D**) demonstrates TB lesions with inflammatory cells, meningitis (upper panel), necrotizing tuberculomas (middle panel) and non-necrotizing tuberculomas (lower panel). These high-power views correspond to panels D, E, I and J from Figure 2.

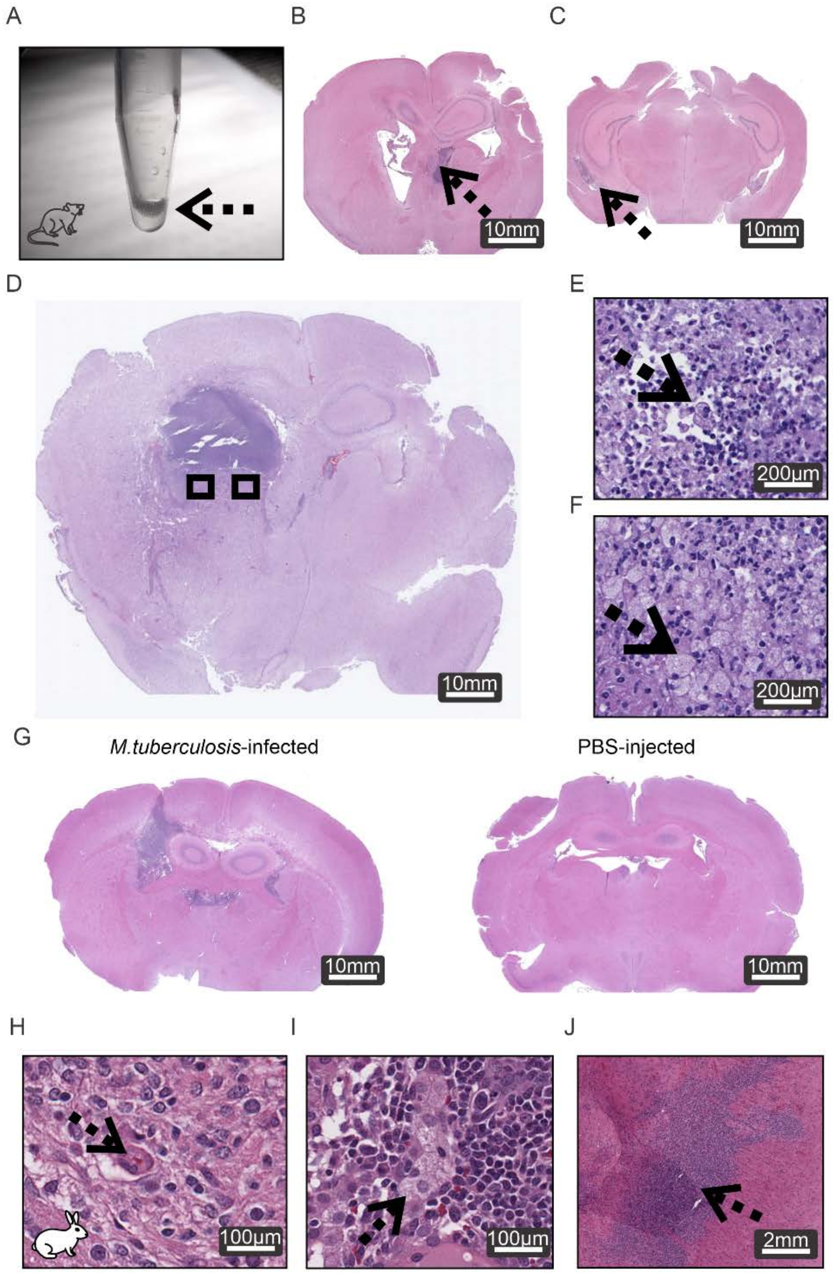

Supplemental Fig. 2. Animal models of TB meningitis. Mice (**A-G**) and rabbits (**H-J**). (**A**) CSF from a *M. tuberculosis*-infected mouse. (**B**) Abundant inflammatory cell infiltration is noted in the fourth ventricle (arrow), choroid plexus and the lateral ventricle (**C**, arrow). (**D**) Necrotic and non-necrotic lesion are noted with scattered multinucleated giant cells (**E**, arrow) and foamy macrophages (**F**). (**G**) Coronal section from a *M. tuberculosis*-infected and control (PBS-injected) mouse 14 days after infection. Similar findings were noted in *M. tuberculosis*-infected New Zealand White rabbits with multinucleated giant cells (**H**), foamy macrophages (**I**) and granulomatous lesions (**J**).

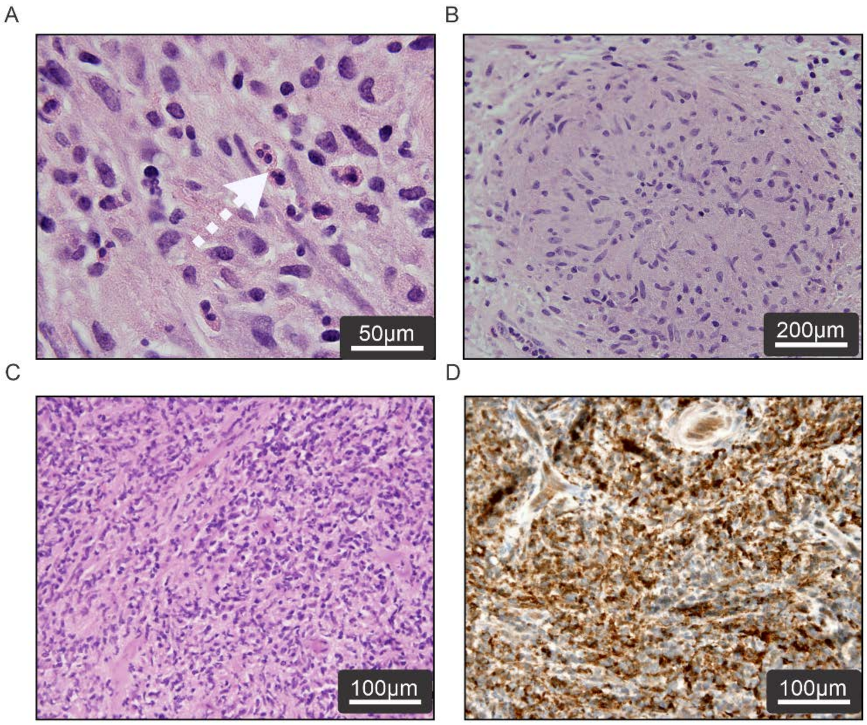

Supplemental Fig. 3. Histopathology in humans with TB meningitis and brain lesions. (**A**) Multinucleated cell (white arrow), (**B**) non-necrotizing granuloma, (**C**) vast mononuclear infiltration, and (**D**) CD68+ cells (brown stain). Panel A-B and C-D correspond to subjects 3 and 5 (Supplemental Table 1) respectively. Panels A and D are high-power views of panels M and N from Figure 2.

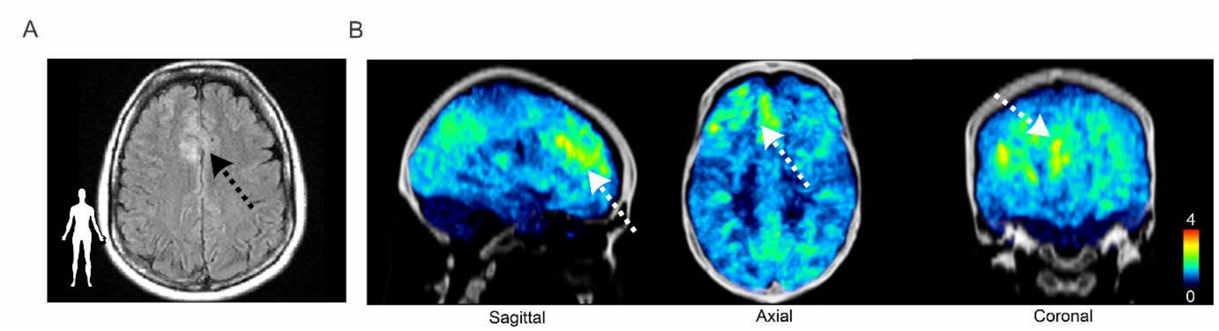

Supplemental Fig. 4. Brain imaging from a patient with TB meningitis. Magnetic resonance imaging (MRI) T2 FLAIR (**A**) and ^{18}F-FDG PET/CT (**B**) from a 40-year-old female with TB meningitis (subject 2, Supplemental Table 1). The arrow points to the TB lesion. PET images are presented as standardized uptake values (SUV). **FLAIR,** fluid-attenuated inversion recovery.

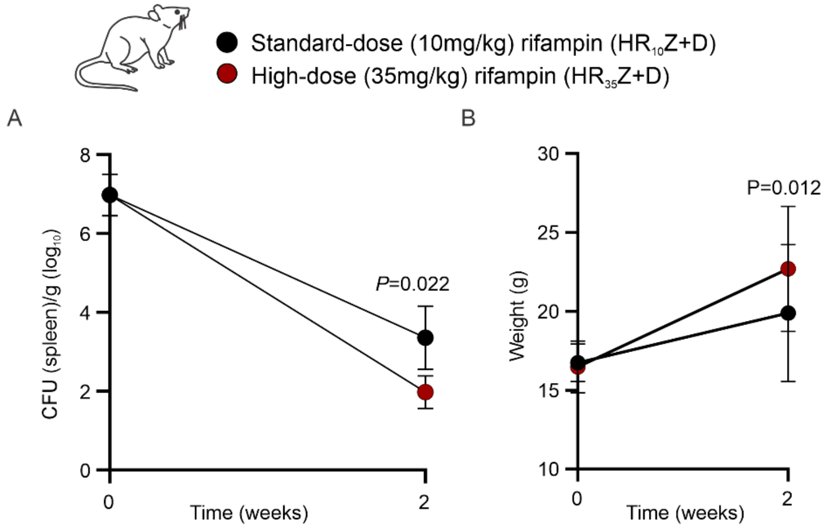

Supplemental Fig. 5. Spleen bacterial burden and weight changes in mice. (A) CFU per gram of tissue in spleen (*n* = 4 animals/group). **(B)** Weight represented in grams (g) (*n* = 10-20 animals/group). Data are represented as mean ± standard deviation. Statistical comparisons were performed using a two-way ANOVA followed by Bonferroni's multiple-comparison test (**A**, **B**).

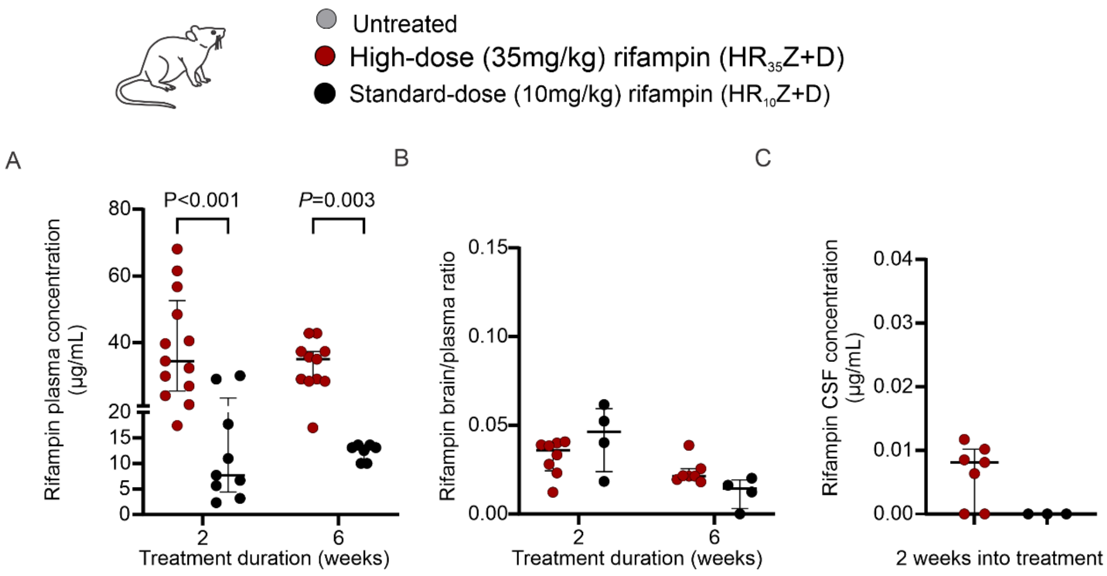

Supplemental Fig. 6. Rifampin tissue concentrations in mice. (**A**) Rifampin plasma concentration (μg/mL) (*n* = 8-13 animals/group per time-point). (**B**) Rifampin brain/plasma ratios (*n* = 4-8 animals/group per time-point). (**C**) Rifampin CSF concentration (μg/mL) at two weeks after initiation of TB treatment (*n* = 3-7 animals/group). Data are presented as median ± interquartile range. Statistical comparisons were performed using two-way ANOVA followed by Bonferroni's multiple-comparison test (**A**).

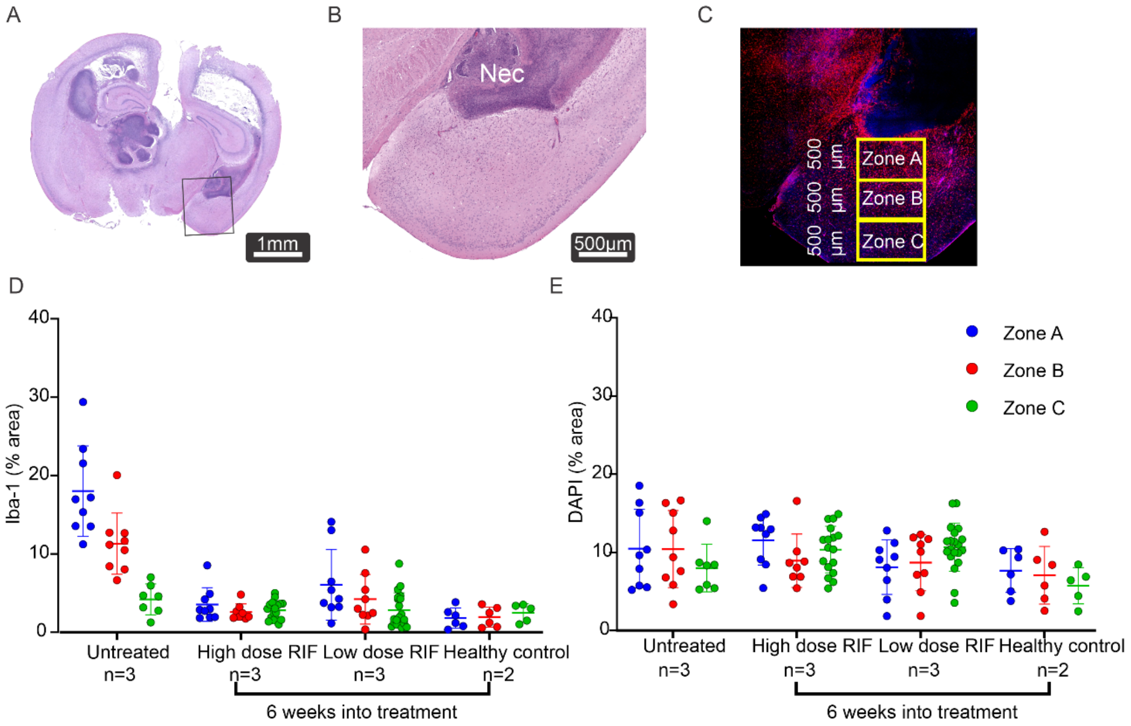

Supplemental Fig. 7. Quantification of immunofluorescence in animal studies. Regions of interest were drawn at the edge of TB lesions to quantify immunofluorescence (Iba-1 & DAPI) at specified locations (zone A, B, C) for all animals and sections. All zones had the same area. (**A-C**) Coronal H&E sections from a *M. tuberculosis*-infected mouse (**A** and **B**) and the corresponding sections used for immunofluorescence of Iba-1 (red) and DAPI (blue) (**C**). (**E-F**) Percentage of Iba-1$^+$ (**D**) and DAPI$^+$ (**E**) cells within zones A, B and C. Data are presented as median ± interquartile range.

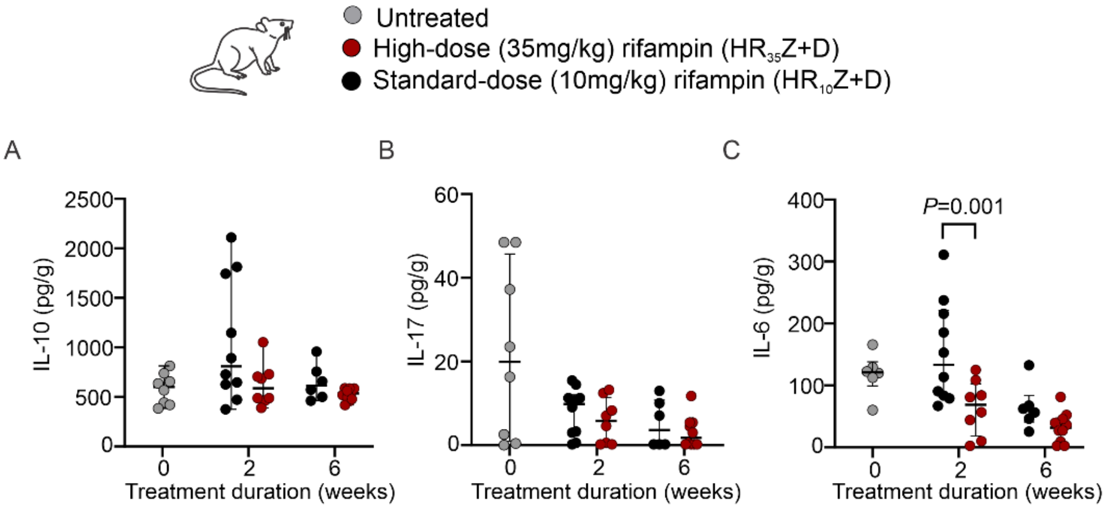

Supplemental Fig. 8. Brain tissue cytokine levels in mice. IL-10 (**A**), IL-17 (**B**) and IL-6 (**C**) levels are shown (n = 3-6 animals/group per time-point, with two technical replicates per animal). Data are presented as median ± interquartile range. Statistical comparisons were performed using two-way ANOVA followed by Bonferroni's multiple-comparison test (**C**).

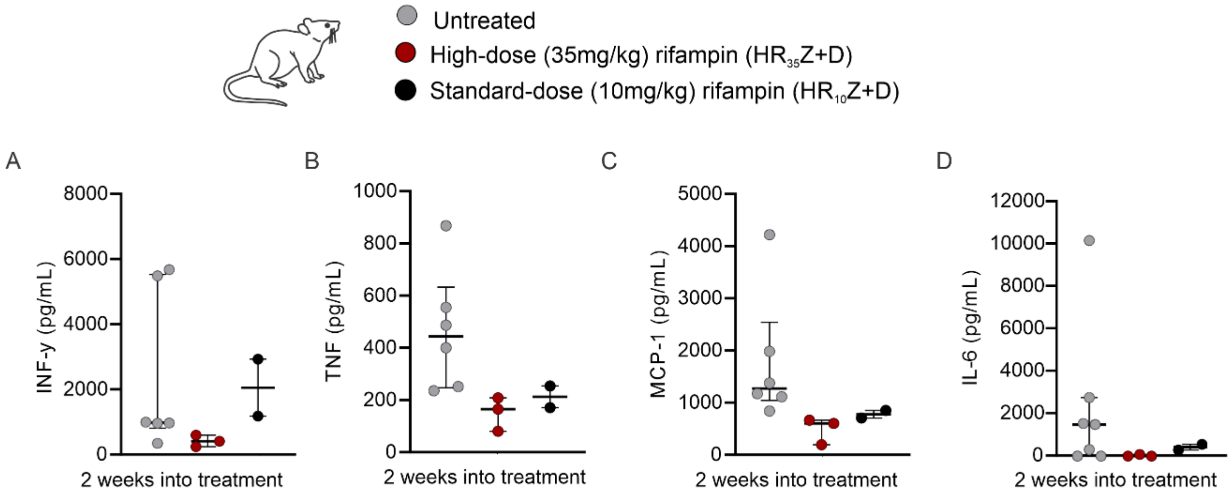

Supplemental Fig. 9. CSF cytokine levels in mice. IFN-γ (**A**), TNF (**B**), MCP-1 (**C**) and IL-6 (**D**) levels are shown (*n* = 2-6 animals/group). Data are presented as median ± interquartile range.

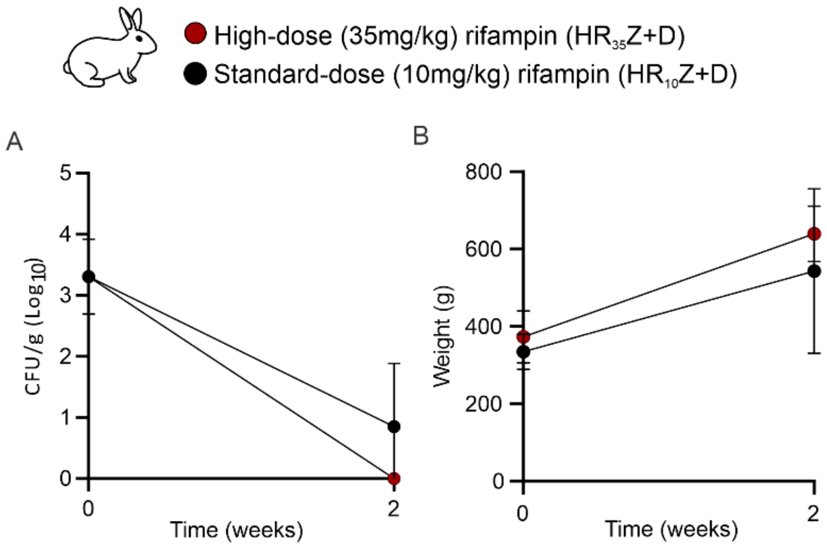

Supplemental Fig. 10. Spleen bacterial burden and weight changes in rabbits. (A) CFU per gram of tissue in spleen (*n* = 3-4 animals/group). **(B)** Weight is represented in grams (g) (*n* = 3-5 animals/group). Data are represented as mean ± standard deviation.

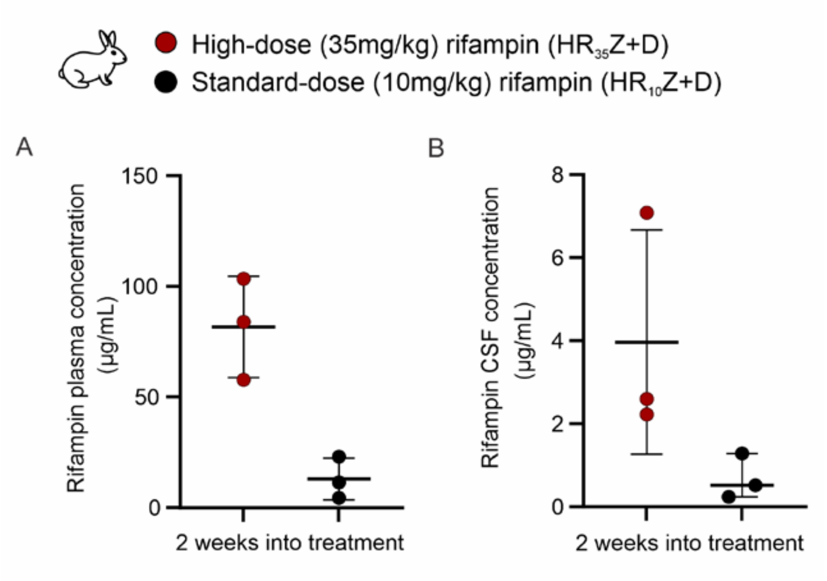

Supplemental Fig. 11. Rifampin tissue concentration in rabbits. Rifampin plasma **(A)** and CSF (**B**) concentration (µg/mL) at two weeks after initiation of TB treatment (*n* = 3 animals/group). Data are presented as median ± interquartile range.

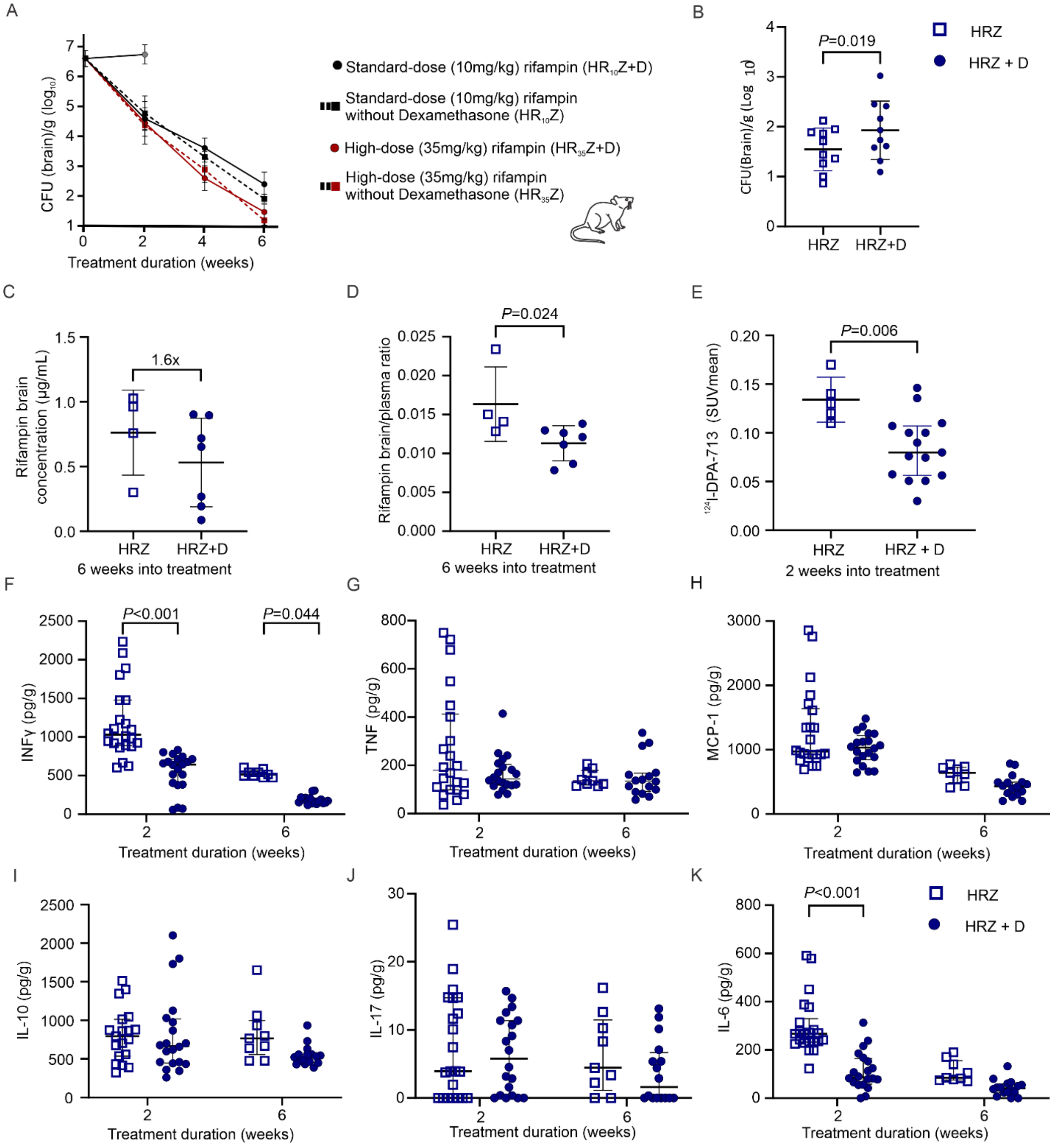

Supplemental Fig. 12. Effect of dexamethasone on rifampin-containing regimens in mice. (**A**) Bacterial burden [colony forming units (CFU) per gram of brain tissue (log_{10})] (*n* = 5 animals/group per time-point). (**B**) Bacterial burden at two weeks after initiation of TB treatment (*n* = 10 animals/group). (**C**) Rifampin brain concentration (µg/mL) (*n* = 4-7 animals/group). (**D**) Rifampin brain/plasma ratios (*n* = 4-7 animals/group). (**E**) Serial ^{124}I-DPA-713 PET imaging presented as SUV_{mean} (*n* = 4-15 animals/group). Brain tissue levels of IFN-γ (**F**), TNF (**G**), MCP-1 (**H**), IL-10 (**I**), IL-17 (**J**) and IL-6 (**K**) (*n* = 4-11 animals/group per time-point, with two technical replicates per animal). Data are presented as median ± interquartile range except for bacterial burden (CFU), which are presented as mean ± standard deviation. Statistical comparisons were performed using a two-tailed Mann-Whitney-Wilcoxon (**B**-**E**) and a two-way ANOVA followed by Bonferroni's multiple-comparison test (**F**, **K**).

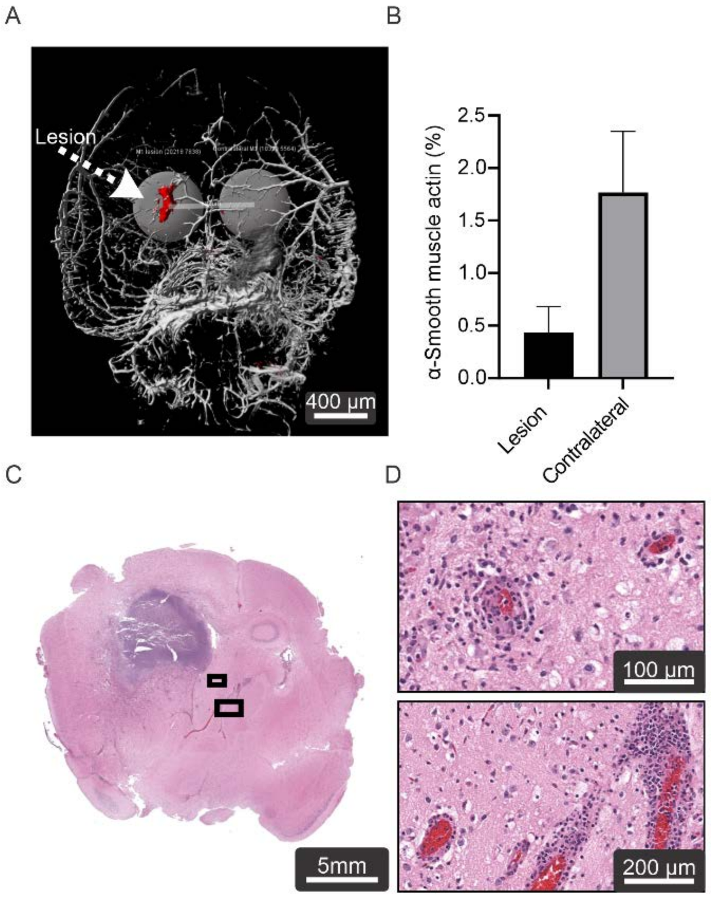

Supplemental Fig. 13. Analysis of blood vessel pathology and vasculitis. (A) Immunostaining of a clarified whole brain (iDISCO) from a mouse with experimentally-induced TB meningitis are visualized in three-dimension and at high-resolution (1 µm): microglia (Iba-1; red) and blood vessels (α-smooth muscle actin; grey). Volumes of interest (VOI) at the site of infection (TB lesions) and the contralateral hemisphere are also shown. **(B)** Quantification of α-smooth muscle actin intensity represented as percentage of area, with lower density representing abnormal, truncated arteries are noted in area with TB lesions. Data from 500 section per animal from two different animals are shown. (**C**) Coronal brain H&E section of a *M. tuberculosis*-infected mouse. (**D**) Transmural inflammation of leptomeningeal arteries. Data represented as median ± interquartile range.

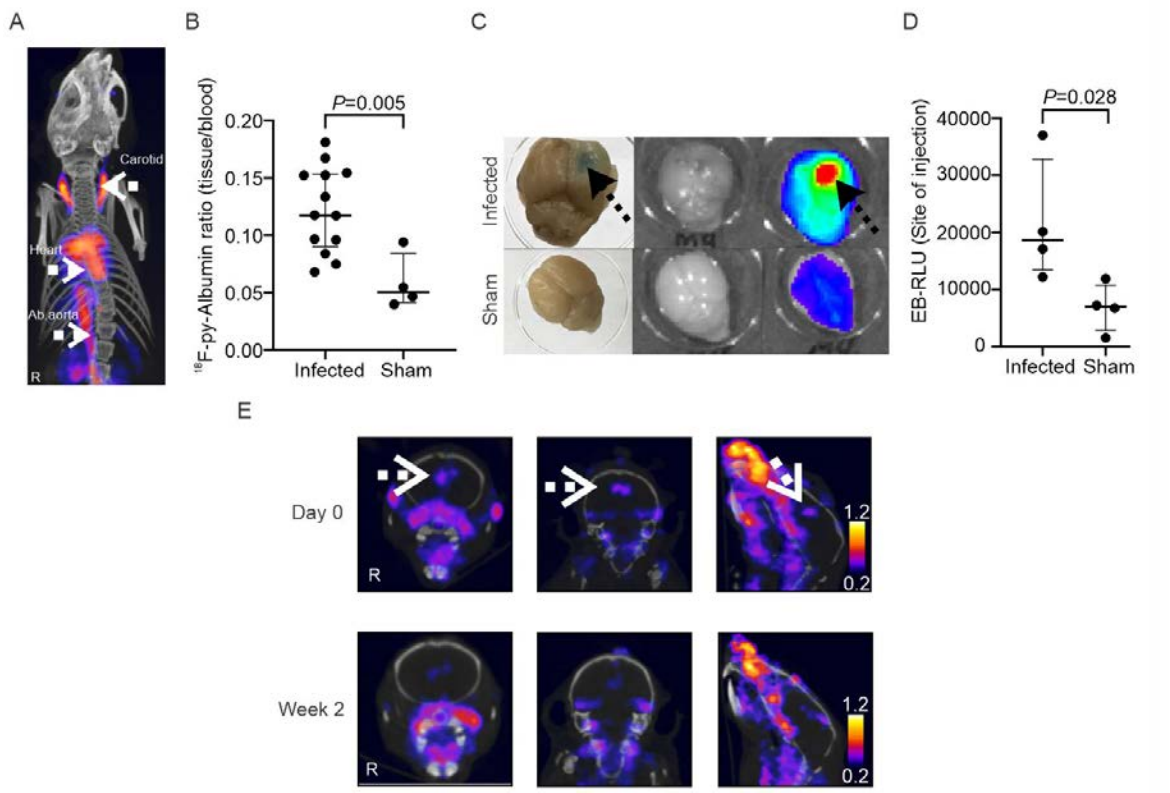

Supplemental Fig. 14. ^{18}F-py-Albumin PET/CT in mice. (**A**) ^{18}F-py-Albumin PET/CT maximum intensity projection (MIP). (**B**) ^{18}F-py-Albumin brain/plasma ratio in *M. tuberculosis*-infected and control (PBS-injected) animals two weeks after infection (*n* = 4-13 animals/group). Whole-brain optical imaging (**C**) and quantification of relative light units (RLU) (**D**) from *M. tuberculosis*-infected and control (PBS-injected) animals injected intravenously prior to sacrifice with Evans Blue (endogenously fluorescent) (*n* = 4 animals/group). (**E**) ^{18}F-py-Albumin PET/CT during TB treatment of *M. tuberculosis*-infected mice (*n* = 9 animals per time-point) with increased signal at the site of TB lesion (white arrow) and changes overtime. Data are presented as median ± interquartile range. Statistical comparisons were performed using a two-tailed Mann-Whitney-Wilcoxon test (**B**, **D**). **Ab aorta**, abdominal aorta.

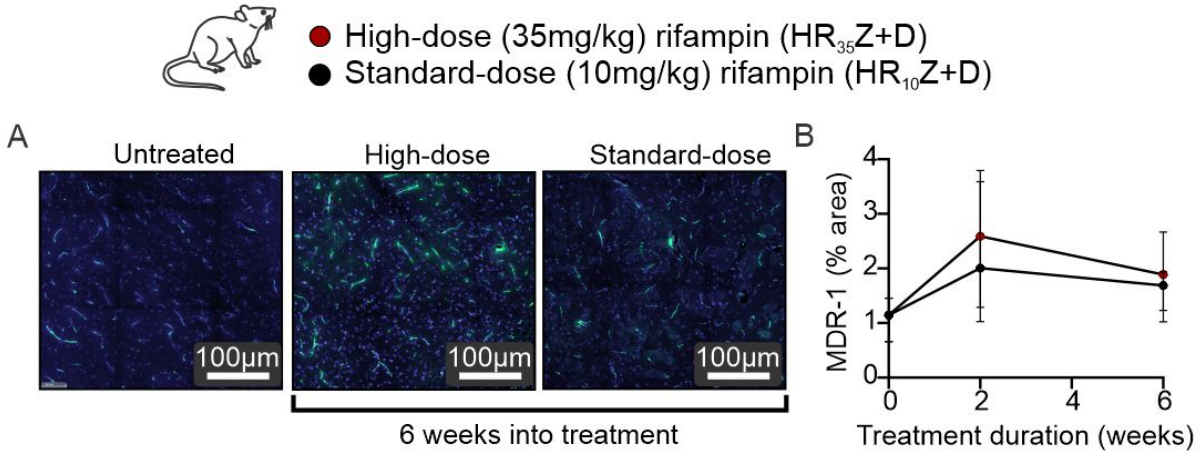

Supplemental Fig. 15. P-glycoprotein expression in mice. (**A**) Representative images from untreated (left panel) animals or animals treated with six weeks of high-dose (middle panel) or standard-dose (right panel) rifampin-containing regimen, demonstrating P-glycoprotein expression (MDR-1 stain in green and DAPI nuclear stain in blue) in brain tissues. (**B**) Quantification of signal (*n* = 3 animals/group per time-point).

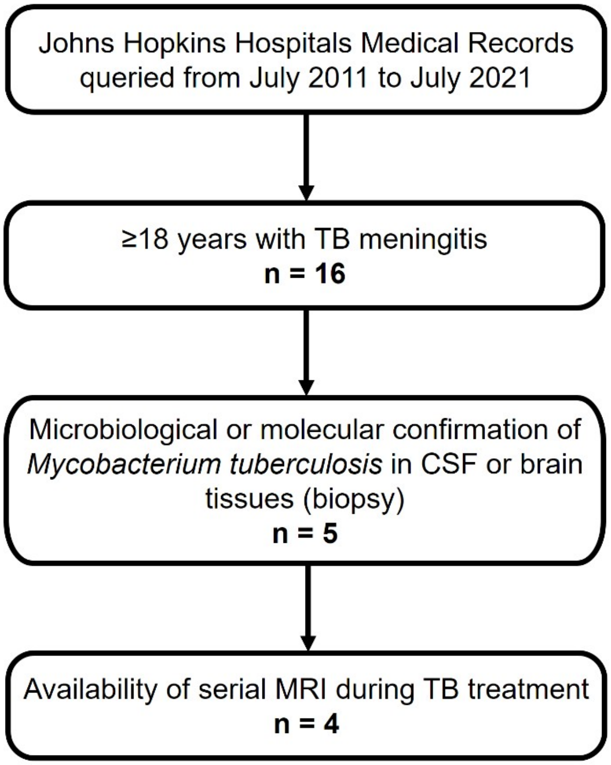

Supplemental Fig. 16. Patient cohort to assess blood-brain barrier disruption during TB treatment. Medical records at the Johns Hopkins Hospitals were queried to identify patients with confirmed with drug-susceptible TB meningitis (microbiology or molecular methods) who underwent serial MRI during TB treatment. Several patients also had brain parenchymal pathology.

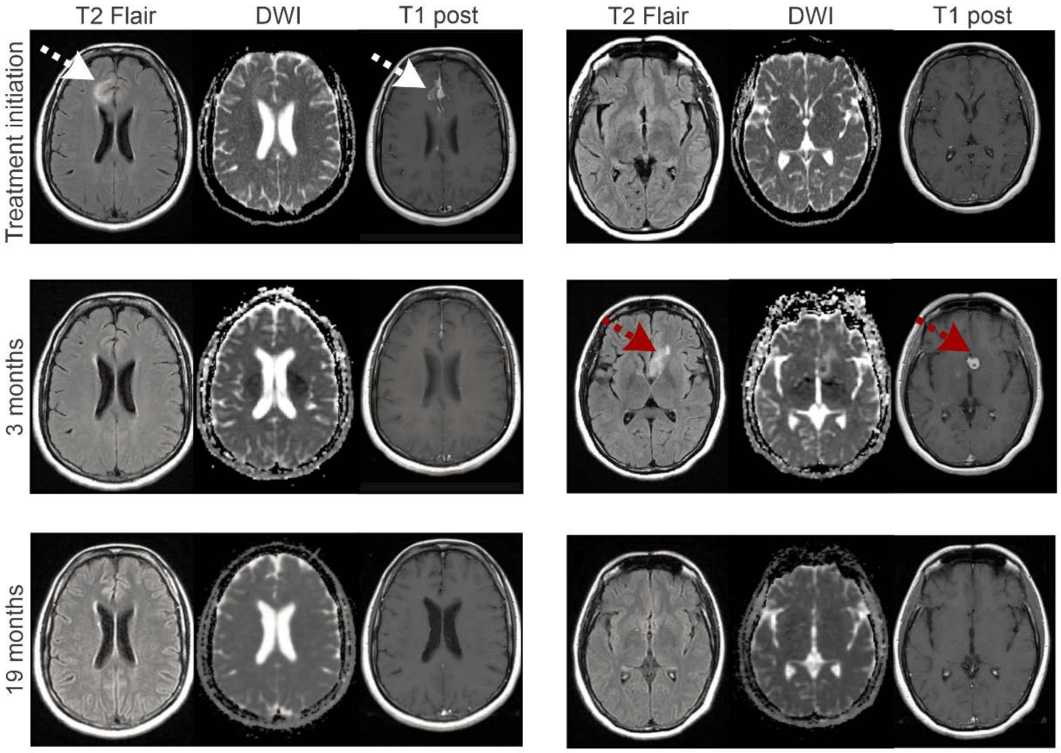

Supplemental Fig. 17. Paradoxical worsening in a patient with TB meningitis. Brain lesion at treatment initiation (white arrow) and new lesion (red arrow) that appeared three months after treatment initiation (subject 2, Supplemental Table 1). The new lesion resolved with continued treatment. Axial sections at two different levels (right and left panels) are shown. MRI from rows 1-2 (right hand panels) correspond to Supplementary Figure 18B. **DWI**, diffusion-weighted imaging; **FLAIR**, fluid attenuated inversion recovery; **T1 post,** T1 post-contrast image.

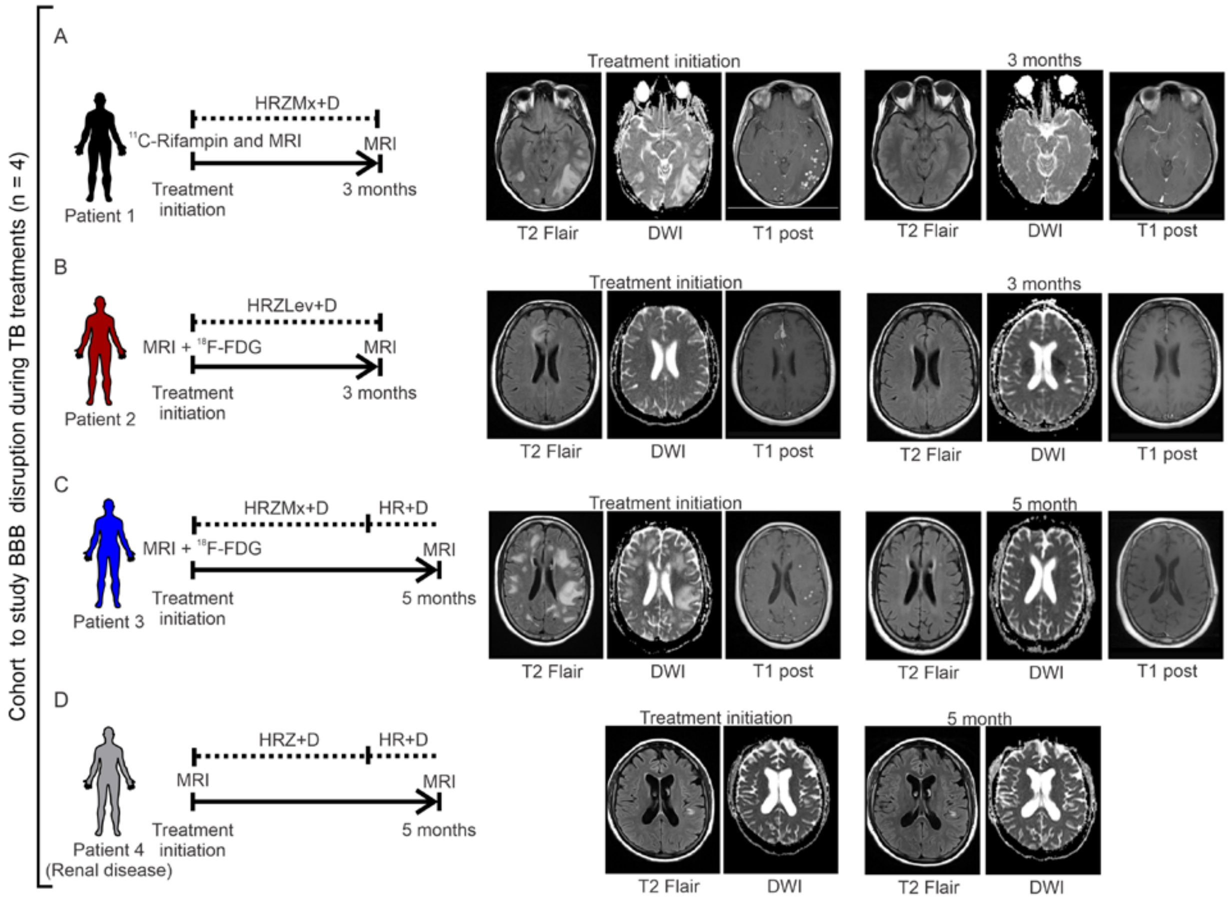

Supplemental Fig. 18. Sequential MRI in patients with TB meningitis. A cohort of patients with TB meningitis who underwent serial MRI during TB treatment was used to assess the blood-brain barrier disruption (*n* = 4 subjects). All patients received 2 months of intensive treatment with HRZ with or without fluoroquinolones, followed by at least 12 months of HR treatment (continuation phase). Panels A and B correspond to Figure 6C and Supplementary Figure 17 (right hand panels; rows 1-2). **ADC**, apparent diffusion coefficient; **D,** dexamethasone; **DWI**, diffusion-weighted imaging; **FLAIR,** fluid-attenuated inversion recovery; **H,** isoniazid; **Lev,** levofloxacin; **Mx**, moxifloxacin; **R,** rifampin; **T1 post,** T1 post-contrast image; **Z,** pyrazinamide.

Supplemental Table 1. Patient cohort to assess blood-brain barrier disruption. Characteristics of patients with TB meningitis used for imaging analysis of blood-brain barrier disruption (T1 post-contrast and apparent diffusion coefficient [ADC]).

**Subject number**	**Gender**	**Age (year)**	**Weight (kg)**	**Diagnosis**	**Confirmatory TB test**	**HIV diagnosis**	**Chest CT**	**Treatment duration at the time of first MRI (days)**
1	F	24	63.5	Tuberculous brain abscess	Culture, Next generation sequencing	Negative	Abnormal	0
2	F	40	57.5	TB meningitis	Culture	Negative	Normal	0
3	F	67	62.8	Tuberculous brain abscess	Culture, biopsy	Negative	Abnormal	0
4	F	34	114	TB meningitis, Chronic renal disease	Culture	Negative	Abnormal	0
**Patients with TB meningitis, but without sequential imaging**								
5	M	54	69	Tuberculous brain abscess	Biopsy	Positive	Abnormal	0

Supplemental Table 2. Drug dosing used for the animal studies. Human equipotent dosing based on (*61*, *62*).

**Drug**	**Human (mg/kg/day)**	**Mouse (mg/kg/day)**	**Rabbit (mg/kg/day)**
Standard-dose rifampin (R_{10})	10	10	30
High-dose rifampin (R_{35})	35	35	105
Isoniazid (H)	10	10	50
Pyrazinamide (Z)	25	150	125
Dexamethasone (D)	0.4	2	1.2

Supplemental Movie 1. Immunostaining of clarified brains. Arteries (α-smooth muscle actin stain in white) and microglia (Iba-1 in red) are visualized in a *M. tuberculosis*-infected mouse brain.
